# Bispidine‐Based Copper(II) Coordination Polymers with Remarkable Dynamic Properties, Selective Volatile Organic Compounds Adsorption, and Exchange Capabilities

**DOI:** 10.1002/chem.202501431

**Published:** 2025-05-27

**Authors:** Meriem Goudjil, Martina Lippi, Chiara Pelosi, Luca Bernazzani, Patrizia Rossi, Paola Paoli, Massimo Cametti

**Affiliations:** ^1^ Department of Industrial Engineering University of Florence via Santa Marta 3 50139 Firenze Italy; ^2^ Department of Chemistry and Industrial Chemistry University of Pisa via G. Moruzzi 13 56124 Pisa Italy; ^3^ Institute for the Chemistry of Organometallic Compounds‐ICCOM Italian National Research Council‐CNR via G. Moruzzi 13 56124 Pisa Italy; ^4^ Department of Chemistry Materials and Chemical Engineering “Giulio Natta” Politecnico di Milano via Luigi Mancinelli 7 20131 Milano Italy

**Keywords:** 1D coordination polymers, bispidine, solvent exchange, structural analysis, VOCs adsorption

## Abstract

This study presents novel bispidine‐based Cu(II) coordination polymers (CPs) with remarkable dynamic properties, volatile organic compounds (VOCs) exchange, and selective adsorption capabilities. The coordination requirements of Cu(II) enable, as demonstrated by SC‐XRD and Powder X‐ray diffraction (P‐XRD), the formation of either 1D ribbon‐like (**1‐TCM^SC^** and **1‐H_2_O^SC^**) or 2D (**1‐MeCN^SC^**) extended frameworks depending on the ability of the trapped solvents to interact as hydrogen bond (HB) donor with the metal's counterion. Hirshfeld Surface (HS) analysis and in situ VT SC‐ and P‐XRD experiments reveal different interchain interactions in 1D *vs* 2D CPs. Solvent exchange experiments on both single crystals (SCs) and microcrystalline samples, set up to evaluate differences in the CPs’ dynamic nature, confirm the drastic effect of CP dimensionality. Additionally, an amorphous desolvated phase **1‐Amorph^Pwd^** was tested for VOC adsorption and demonstrated to display affinity for acetonitrile and nitromethane, with high selectivity for the latter, but also to have the ability to trap aromatic VOCs, capturing up to ca. 2 mmol/g of solvent. The adsorption experiments, conducted at *r*.*t*., 1 atm, and no prior activation, underscore the potential of these materials for environmental and industrial applications. This work emphasizes the unique dynamic and selective behavior of bispidine‐based CPs and provides a foundation for their scalability and practical implementation in VOCs capture technologies.

## Graphical abstract

Novel bispidine‐based Cu(II) CPs show remarkable selective VOC adsorption and 1D to 2D transformations.

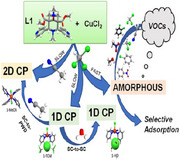

## 1 Introduction

A rapidly evolving field in chemical science is represented by research on coordination polymers (CPs), which are materials characterized by the assembly of metal ions and organic ligands into extended networks. CPs are notable for their structural diversity and potential applications in areas such as gas storage, catalysis, and selective adsorption. They can be effectively distinguished based on their dimensionality. Indeed, CPs can be categorized as 1D, 2D or 3D, depending on the prevalent direction of the extension of the metal‐ligand interactions. This characteristic not only defines the structural framework of the polymers but often dictates their final properties and potential applications. While 2D CPs, which feature layered structures that extend in two directions, are particularly useful in electronic, catalysis, and sensing applications,^[^ ^1^, ^2^ ^]^ 3D CPs are often constituted by frameworks embedded with large open pores or channels.^[^ ^1^, ^3^ ^]^ For this reason, they are widely studied for gas storage, adsorption, and separation due to their high porosity and stability.^[^ ^3^ ^]^ Not surprisingly, most of the popular metal organic frameworks (MOFs) are often 3D CPs. 1D CPs, on the other hand, are characterized by simpler chain‐like structures and, until recently, they were never considered promising for adsorption applications, as they are often devoid of large internal pores or channels. A significant change in perspective can be obtained, in our view, by reviewing the initial works by Takamizawa's (and others),^[^ ^4^ ^]^ where linear 1D CPs, although lacking apparent voids within their chains, were shown to be able to adsorb a series of different volatile guests by “making space” for them within their framework. Inspired by those initial reports, we have developed a class of novel 1D CPs based on bispidine ligands. Bispidines are bicyclic molecules, easily synthesized, and highly structurally tunable, which are commonly used as metal binders.^[^ ^5^ ^]^ Over the last few years, we were engaged in the use of variously functionalized bispidine ligands to form 1D CPs and in the exploration of their adsorption properties.^[^ ^6^ ^]^ Made of parallel, loosely interacting chains of various topologies (linear, zig‐zag, ribbon‐like, helical, and poly‐catenane), these CPs had shown marked abilities to adsorb volatile organic compounds (VOCs) from the atmosphere in amounts of the order of 1–3 mmol/g. Upon adsorption, they undergo a marked reorganization in chains orientation driven by the necessity to accommodate the incoming volatile guests.

**Scheme 1:**
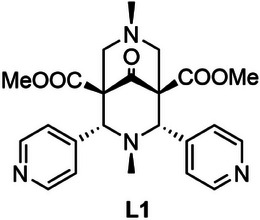
Molecular formula of bispidine ligand **L1**.

In this work, we have studied CPs made by the combination of ligand **L1** (see Scheme 1) and CuCl_2_ in order to evaluate their dynamic properties in terms of solvent adsorption and exchange. The role of the metal center can be crucial and, therefore, the use of Cu(II) in this work, never tested before with bispidine‐based CPs, could open the way to novel types of frameworks and dynamic properties. Firstly, we have explored the possibility to obtain novel CPs in the form of single crystals (SCs) which were then fully characterized by SC‐XRD. Also, we tested their capability to undergo solvent exchange processes, as the single‐crystal‐to‐single‐crystal (SC‐to‐SC) fashion is particularly appealing to gather detailed structural information of the process. On this regard, we have obtained three different species in the form of SCs. Two of them, **1‐TCM^SC^** and **1‐MeCN^SC^** of composition [Cu(**L1**)_2_Cl_2_·(TCM)_4_] and [Cu(**L1**)_2_Cl_2_·(MeCN)_2_], respectively, were obtained by a three‐layer crystallization method (SI) and additionally characterized by VT SC‐XRD and hot‐stage microscopy (TCM = Chloroform; MeCN = Acetonitrile). **1‐TCM^SC^** is a 1D CP with a ribbon‐like topology, although with a particular coordination geometry around the Cu(II) center, never observed in previously reported bispidine‐based 1D CPs. On the other hand, **1‐MeCN^SC^** is a 2D CP and it displays a completely different pattern of metal‐ligand interactions. The third CP, named **1‐H_2_O^SC^**, of composition [Cu(**L1**)_2_Cl_2_·4.65(H_2_O)] resulted from a SC‐to‐SC transformation starting from **1‐TCM^SC^**. Molecular packings were studied by Hirshfeld surface (HS) analysis. **1‐TCM^SC^** and **1‐MeCN^SC^** were then exposed to vapors of a series of common aromatic and aliphatic VOCs (MeOH, EtOH, MeCN, MeNO_2_, dicholoromethane (DCM), nitrobenzene (NB), chlorobenzene (ClBz), and 1,2‐dichlorobenzene (DCB)), to test their dynamic nature and gather essential structural information. After that, we have put effort into producing **1‐TCM^SC^** and **1‐MeCN^SC^** CPs in the form of microcrystalline powders and testing them for solvent exchange transformation. Attempts to obtain **1‐TCM^SC^** in the form of microcrystalline powders, led to a desolvated amorphous phase **1‐Amorph^Pwd^**, which was then an optimal candidate for adsorption studies. All results were interpreted in light of the dynamic nature of 1D CPs vs 2D frameworks, due to the underlying weak inter‐chain interactions compared to what occurs in layered structures. In addition, the role of the combination of Cu(II) adaptability and the diverse solvent hydrogen bonding (HB) donor capabilities has been brought about as a critical element for the dimensionality of the CP observed.

As VOCs can significantly impact air quality and human health, their adsorption is of particular interest for environmental applications.^[^ ^7^ ^]^ Hence, research on novel materials to be used as VOC adsorbents is increasingly recognized, especially if selectivity is achieved, as this work demonstrates.

## 2 Results and Discussion

### 2.1 SC X‐ray Structures

**Figure 1:**
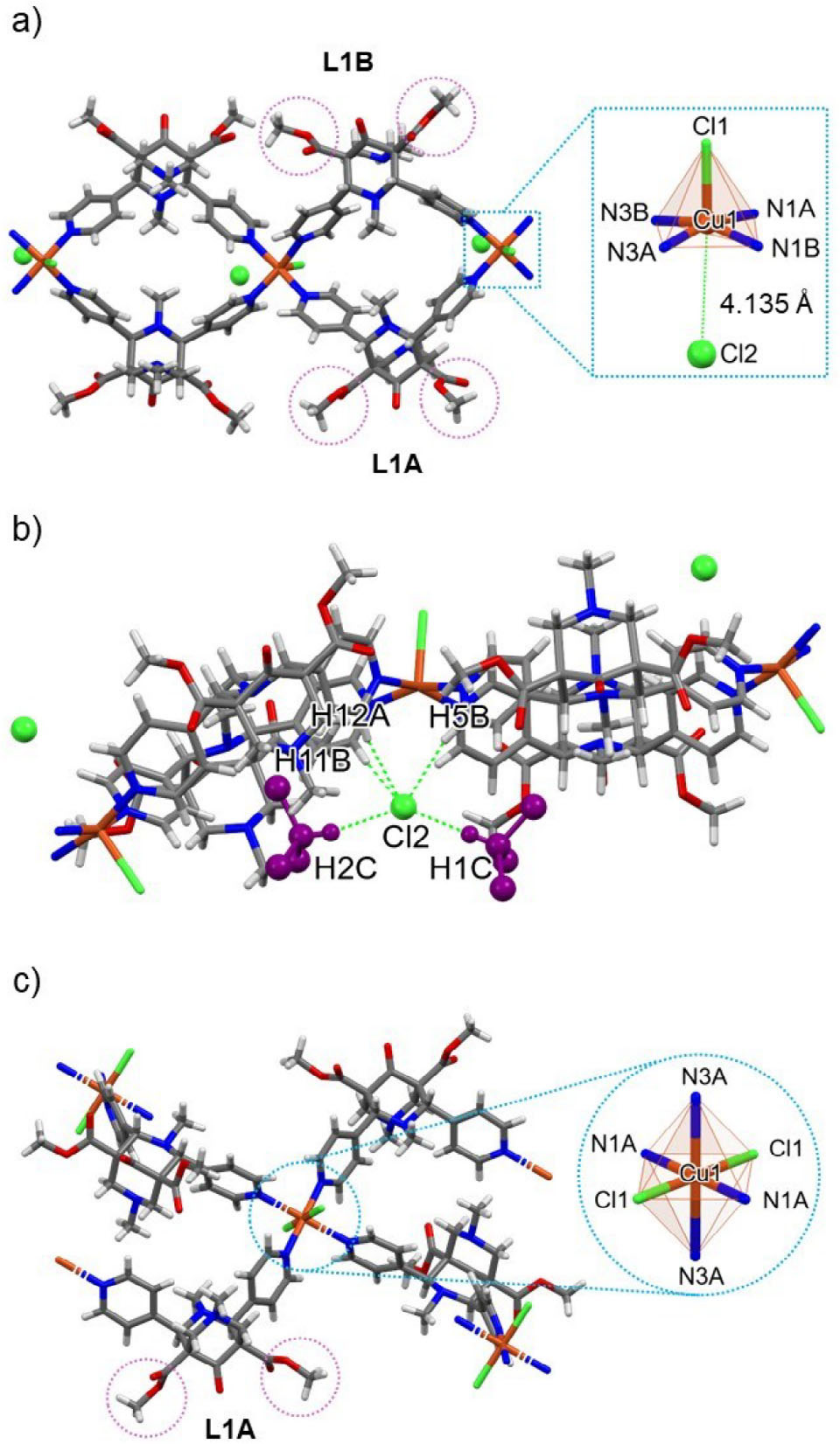
View of the ribbon motif in **1‐TCM^SC^** illustrating the coordination of the Cu(II) center and the orientation of L1A/L1B‐ester groups arranged in a *syn* mode (a). View showing the intermolecular interactions between Cl2 anion with the framework and two TCM molecules (drawn in purple, the other two were omitted for clarity) (b). Representation of the coordination environment around Cu(II) and the *anti*‐oriented ester groups in **1‐MeCN^SC^** (c). Color code: C: gray; H: white; N: blue; O: red; Cu: orange; Cl: green. Cl2 ions are depicted in Ball&Stick representation. Solvent molecules in (a) and (b) were omitted for clarity.

**Figure 2:**
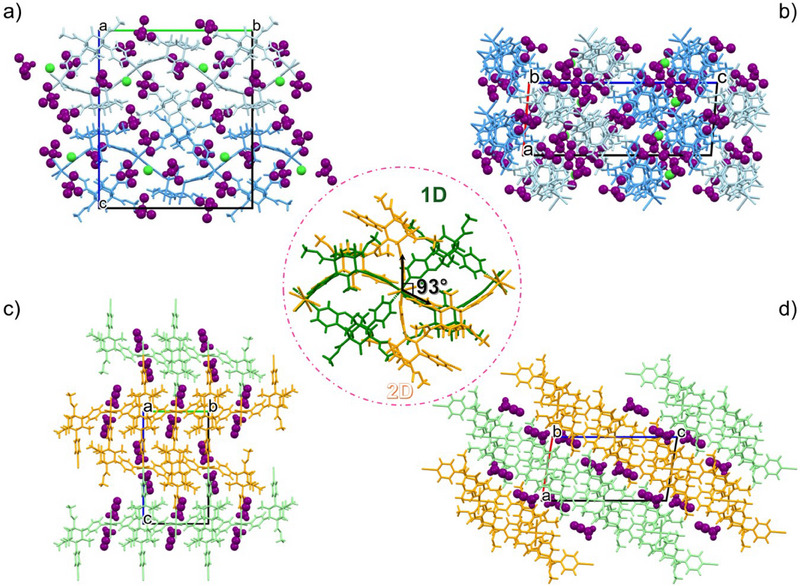
The single‐crystal structures of **1‐TCM^SC^** and **1‐MeCN^SC^** CPs exhibiting distinct topologies: view of the 1D ribbon chains and TCM molecules in **1‐TCM^SC^** seen along the [100] (a) and [010] (b) directions. Packing modes of the 2D polymeric framework of **1‐MeCN^SC^** within MeCN molecules along the [100] (c) and [010] (d) directions. Inset of the structures overlay highlighting the motif formed by the four **L1** bispidine ligands coordinating the Cu(II) center, arranged in ribbon‐units in **1‐TCM^SC^** (green framework) and positioned 93° apart in **1‐MeCN^SC^** (yellow framework) (b). TCM and MeCN molecules are drawn in Spacefill representation and colored purple. Color code: C: gray; H: white; N: blue; O: red; Cu: orange; Cl: green.

Dark‐blue prismatic SCs of **1‐TCM^SC^** and green tubular ones of **1‐MeCN^SC^** were obtained by using a slow crystallization technique involving three different solvent layers (see SI), and their crystal structures were determined by SC‐XRD characterization (see Table S1). The structural analysis revealed a 1D CP with a wavy ribbon‐like topology for **1‐TCM^SC^**, with 2:1 ligand:metal units extending into non‐interpenetrated wavy chains along the [100] direction (Figure 1a and Figure S1a). Its asymmetric unit consists of one Cu(II) cation, two **L1** ligands (labelled as L1A/L1B), in a classic *chair‐chair* conformation and with the ester groups arranged in a *syn* mode (as shown in Figure 1a), two Cl^−^ ions, and four TCM molecules. The main difference with respect to previously reported ribbon‐like bispidine‐based CPs, such as those having Mn(II) as the metal center^[^ ^8^ ^]^ is related to the Cu(II) coordination. Indeed, the Cu(II) ion can be better described as penta‐coordinated in a slightly distorted square pyramidal geometry (Addison trigonality index^[^ ^9^ ^]^ τ^5^ = 0.08), rather than an octahedral one (as the Mn(II)), as it coordinates with a chloride ion (Cl1) in the apical position at a distance of 2.469(1) Å and with four N‐pyridinic atoms from four **L1** molecules in the equatorial plane (Cu—N distances ranging from 2.036(2) to 2.063(2) Å (Table S2)), see Figure 1a. The second chloride (Cl2) instead lies in the opposite direction with respect to the Cu─Cl1 bond but is far too distant to be considered coordinated with the metal ion (Cu─Cl2: 4.135(1) Å). Examples where chloride ions do not exhibit direct coordination to the metal center have been documented in various compounds, including molecular complexes with pyrazol‐derivatives containing Cu(II)^[^ ^10^ ^]^ or Zn(II), and bispidine ligands with Mn(II), Fe(III), Cu(II), Zn(II), and Pd(II), as well as heteronuclear bispidine complexes with Cu(II)/Zn(II).^[^ ^6^, ^11^ ^]^ In the present case, the uncoordinated chloride ion is involved in CH···Cl^−^ HBs^[^ ^12^, ^13^ ^]^ both with the CH_arom_ donors provided by the pyridine moieties of the ligand and the two neighboring TCM molecules (Figure 1b and Table S2 in Supporting Information). Another significant structural feature that distinguishes **1‐TCM^SC^** from all the previously reported Mn(II)‐based CPs containing chloroform is related to the symmetry of the macrocyclic units (the latter defined by two contiguous metal coordination ‐M<(**L1**)_2_>M‐ repeating ribbon units). In the Mn(II)‐based CPs, the macrocyclic cavity possesses a center of symmetry or a pseudo center of symmetry as opposed to a pseudo‐symmetry plane passing through the Cu─Cl bonds in **1‐TCM^SC^**.^[^ ^14^ ^]^ Hence, the different relative arrangement and related steric hindrance of the substituents on the bispidine nitrogen atoms which overlook the macrocyclic cavity: for example, in **Mn(L1)_2_Cl_2_‐2TCM^SC^** (RIMYAQ)^[^ ^6g^ ^]^ the distances between the carbon atoms of the ‐NCH_3_ groups are about 6.5 Å vs 6.4 Å and 9.5 Å in **1‐TCM^SC^**, respectively. We can speculate that this arrangement allows for the partial insertion within the macrocyclic cavity of the TCM molecule less tightly bound to the chloride anion Cl2, notwithstanding the smaller length of the macrocyclic cavity in **1‐TCM^SC^** as estimated by the distance separating the metal centers (11.8 Å vs. 12.8 Å in **Mn(L1)_2_Cl_2_‐2TCM^SC^**). The noticeably shorter M—N distances in **1‐TCM^SC^** (2.036‐2.064 Å vs. 2.330‐2.353 Å in **Mn(L1)_2_Cl_2_‐2TCM^SC^**) provide an explanation for this latter observation and suggest a greater crowding around the copper ion for the same 1D ribbon motif (see Figure 2a,b) as also evidenced by the distances between the centroids of the adjacent pyridine rings. By contrast, the Cu—Cl distance (2.469 Å) fits in the range of Mn—Cl distance (2.460‐2.485 Å), and in both cases each metal‐bound chloride ion interacts with a TCM molecule (Table S2 in Supporting Information). These abovementioned differences, including the greater versatility in the coordination mode of Cu(II) compared to Mn(II), can be directly linked, whether they are the cause or simply the effect, to the large amount of solvent present in the framework of **1‐TCM^SC^**: four TCM molecules per metal ion as compared to two solvent units in the Mn‐based CP (RIMYAQ^[^ ^6g^ ^]^). By virtual removal of the TCM molecules in **1‐TCM^SC^**,^[^ ^15^ ^]^ pocket‐shaped voids with narrow inter‐pockets connections appeared (Figure S1b,c in Supporting Information) which represent ca. 35.8% of the total unit cell volume. Additional CH···O interactions which primarily contribute to inter‐ribbon interactions (Table S3 in Supporting Information), further contribute to stabilize the crystal packing (for a detailed description refer to Supporting Information, Section S2 and Figures S1,S2). Interestingly, an isostructural species, named **1‐H_2_O^SC^** hereafter, which has TCM replaced with water molecules, has been obtained from a solvent‐exchange experiment carried out on **1‐TCM^SC^** (details in the Section S2 in the Supporting Information) via a SC‐to‐SC transformation. In particular, in the resulting crystal lattice two out of the seven co‐crystallized water molecules (4.65 per metal ion if the real occupancy is taken into account) take the place of the two TCM molecules bound to the chloride anion (see Figure S3a), the latter being still far distant from the copper ion, Cu···Cl2: 4.051(2) Å (Table S2), which maintains the square pyramidal geometry, with τ^5^ = 0.12 (a detailed description of **1‐H_2_O^SC^** and its crystal packing is reported in the Supporting Information, Section S2.2, see Figures S3‐S5 and Table S4). This finding suggests that the 1D structures with ribbon‐like topology of the CP together with the paired counterion are quite stable toward the TCM‐H_2_O exchange, allowing the loss of the TCM molecules and the uptake of the water ones without noticeably changing (Figure S3). However, the significant variation in the unit cell dimensions and space group which accompanies the solvent exchange leading from **1‐TCM^SC^** to **1‐H_2_O^SC^** suggests an important reorganization of the crystal lattice. Indeed, we must consider that the TCM molecules are initially hosted in pockets joined through narrow connections, which implies that contiguous CP chains should move apart to allow their release (see Figure S5). This chain mobility should not come as a surprise when dealing with highly dynamic 1D CPs like the ones described here. Moreover, **1‐H_2_O^SC^** demonstrated greater stability than **1‐TCM^SC^**, as its transformation remained irreversible even after exposure to chloroform vapors for up to 2 weeks.

SC‐XRD analysis of **1‐MeCN^SC^**, obtained by replacing MeOH with MeCN in the three‐layer crystallization procedure, revealed a completely different situation. Although metal‐ligand stoichiometry is maintained, metal coordination does not lead to a 1D ribbon‐like motif as in **1‐TCM^SC^**, but rather to an extended network (see Figure 1c), with the bispidine ligands oriented approximately 93° apart relative to each other, thus developing a 2D polymeric structure (see Figure 2, central inset). In the asymmetric unit, there are one‐half of Cu(II) ion (which sits on an inversion center), one **L1** molecule with the ester groups oriented in *anti‐*conformation (Figure 1c), one chloride ion and one MeCN solvent molecule. The Cu(II) ion is hexacoordinated by two chloride ions at 2.344(3) Å and two pyridinic nitrogen atoms at 2.027(1) Å (see Tab. S2) in the equatorial plane; the apical positions are occupied by two N‐atoms bound at a longer distance Cu—N3A: 2.489(2) Å (Table S2) completing a Jahn‐Teller tetragonally distorted octahedron^[^ ^16^ ^]^ (shown in Figure 1c), with the tetragonality being 0.88. The MeCN molecules are incorporated into the crystal lattice (Figures 2c‐d), occupying large, isolated voids that account for 13.7% of the total unit cell volume (Figure S6c). The MeCN molecules come in contact with the CP through a CH···N interaction (Table S5, Supporting Information) which occurs between the nitrile functional group and the bispidine aromatic CH (C10AH10A···N1AC, 2.619(2) Å/140(1)° see Figure S6b). Mutual CH···O interactions involving the bispidine ligand (listed in Table S5) contribute to strengthening the crystal packing (for a detailed description refer to Supporting Information, Figure S6a).

### 2.2 HS Analysis

HS analysis was used to visualize and get a quantitative picture of the intermolecular interactions in the packing with a special focus on those involving the solvent guests. As to **1‐TCM^SC^**, the four independent solvent molecules experience different environments, while chloride ion Cl2 does not appear to be involved in any metal‐Cl interaction. In fact, the five red spots which characterize the HS of Cl2 suggest its involvement in five H‐bond interactions: with two HB donating TCM molecules (see Supporting Information, Table S3, Entries:11,12) and with the CP belonging to the same ribbon via the ‐CH groups provided by three pyridine rings (Table S3. Entries: 8–10). In turn, the two chloride‐bound (TCM) molecules experience a different environment in terms of interactions (one red spot for the TCM molecule more tightly bound to the chloride ion; two almost identical less evident red spots for the other, which suggest a further interaction with an ‐NMe group provided by the array ligand, Table S3, Entry: 15) and their related fingerprint plots (FPPs) (Figure S7). A third H‐bond donating TCM molecule interacts with the Cu‐coordinated chlorine ion Cl1 (Table S3, Entry: 7), the most evident red spot, and is further held in place by an OMe···Cl interaction (Table S3, Entry: 16) with the H‐bond donor provided by an adjacent ribbon. The same OMe group weakly binds the fourth independent TCM (Table S3, Entry: 17) which in turn is strongly interacting (a well‐evident red spot) via a CH···O═C HBs provided by the same ribbon (Table S3, Entry: 14). As for the CP, the most important inter‐ribbon interaction, based on the intensity of the red spots, is that involving the carbonyl oxygen atom of one ester group (C20B = O5B) which works as acceptor toward a CH donor provided by the bispidine scaffold of an adjacent ribbon (Table S3, Entry: 3) thus originating a double interaction between contiguous ribbons.

The HS of MeCN in **1‐MeCN^SC^** well evidences the propensity of this molecule to act essentially only as an H‐bond acceptor via the electron‐rich region of the C≡N group (Table S5, Entry: 4). The related fingerprint plot reveals that the N···H interactions contribute for about the 31% to the HS (Figure S8). By contrast, the CP appears more prone to intermolecular interchain interactions, as highlighted by the more numerous and evident red spots characterizing the corresponding HS. Accordingly, the contribution of the O···H interaction to the CP HSs, as evaluated by the related FPPs, equals to 19.6% in **1‐MeCN^SC^** and 14.8% in **1‐TCM^SC^**. This result is not unexpected given that in the TCM solvated CP there are plenty of species, including the chloride anion, able to engage most of the potential H‐bond donors and acceptors of the CP framework, thus limiting the CP···CP interactions. Structural investigations, supported by HS, thus suggest a significantly more dynamic behavior of **1‐TCM^SC^** compared to **1‐MeCN^SC^**, which features a more rigid (2D vs. 1D framework) and efficiently packed crystal structure. The data suggests that the weaker interchain interactions in **1‐TCM^SC^** result in a looser packing arrangement. This, in turn, enhances the flexibility and mobility of the 1D chains, potentially promoting efficient solvent exchange and contributing to the dynamic nature of the system. In contrast, the reorganization capability of the entire 2D framework in **1‐MeCN^SC^** is considerably more limited, reflecting its higher structural stability during guest exchange or removal (vide infra) due to the stronger contribution of the intermolecular interactions reinforcing the entire framework.

### 2.3 VT SC‐XRD Characterization

To investigate phase stability and potential structural transformations (e.g., phase transitions) in SCs of **1‐TCM^SC^** and **1‐MeCN^SC^**, in situ SC‐XRD experiments were conducted at variable temperatures by heating the SC sample gradually from 100 to 400 K, with measurements taken at different temperature increments (10‐50K).

The thermal expansion coefficients (TECs)^[^ ^17^ ^]^ have been calculated from changes in linear parameters (α) and volume (β) using cell parameter values at 100 K as the reference. Table S6 and Table S7 report the TECs for **1‐TCM^SC^** and **1‐MeCN^SC^**, respectively. In the case of the **1‐TCM^SC^**, the analysis of the temperature dependence on the lattice constants revealed a consistent trend of isotropic expansion up to 400 K (see Table S6), accompanied by an increase in the unit cell volume. Surprisingly, even when the temperature exceeded the boiling point (b.p.) of TCM (334 K), no evaporation occurred, and the same quantity of TCM molecules remained preserved within the framework, as inferred from the analysis of their site occupancy factors. Overall, both the crystal morphology and structure of this CP remained intact throughout the examined temperature range. The examination of the TECs coefficients for **1‐MeCN^SC^** indicated a narrow isotropic thermal expansion between 100 and 400 K (see Table S7), along with an increase in the unit cell constants. Throughout the explored temperature range, the crystal shape and framework of this CP were also preserved. Similar to the solvent behavior observed in **1‐TCM^SC^**, MeCN in **1‐MeCN^SC^** did not evaporate after exceeding its b.p. of 355 K, and the solvent sites remained fully occupied. These results demonstrate the high stability of both frameworks up to 400 K.

### 2.4 Hot‐Stage Microscopy

Crystals of **1‐TCM^SC^** exhibit remarkable stability when heated from 298 K to 403 K, as shown in Figure S9, consistent with SC‐ and Powder X‐ray diffraction (P‐XRD) data (Section S5). Significant changes in morphology and color were observed between 408–428 K, during which the crystals expanded in size and turned yellow. At 433 K, the crystals began to melt, with complete melting occurring at 443 K. During the cooling cycle, conducted at the same rate (see Experimental section), the yellow liquid solidified as a powder at approximately 423 K. Hot‐stage micro‐images of **1‐MeCN^SC^** were also recorded (Figure S10) When heated from room temperature to 408 K, the crystals of **1‐MeCN^SC^** retain their morphology, transitioning in color from green to yellow at 408 K. As the temperature increased, the crystal sample gradually turned red/brown, becoming a vitreous block, partially melted at 443 K. Upon cooling, the sample solidified into a red/brown block.

### 2.5 Adsorption and Exchange Experiments

**Figure 3:**
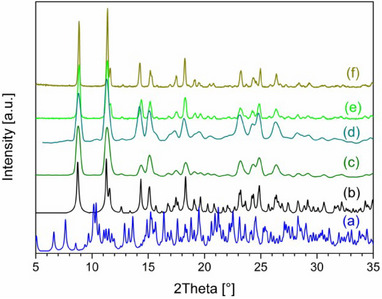
P‐XRD data: simulated patterns of **1‐TCM^Pwd^** (a), and **1‐MeCN^Pwd^** (b); experimental patterns of products from SC‐to‐Pwd transformations of **1‐TCM^SC^** exposed to MeCN (c) and MeNO_2_ (d) vapors for 24 hours and 3 weeks, respectively; and experimental patterns of **1‐Amorph^Pwd^** subjected to MeCN (e) and MeNO_2_ (f) vapors over 2 weeks. Patterns (c)‐(f) match the simulated pattern of the **1‐MeCN^Pwd^** phase.

Having in mind the potential of this class of 1D CPs as materials for adsorption application, we started evaluating their dynamic behavior by exposing SCs of **1‐TCM^SC^** toward common VOCs of interest, such as MeOH, EtOH, MeCN, MeNO_2_, dicholoromethane (DCM), nitrobenzene (NB), chlorobenzene (ClBz) and 1,2‐dichlorobenzene (DCB). Experiments on SC can be extremely important as they offer precise structural information, enabling detailed studies of adsorption sites and mechanisms, without the interference of defects or grain boundaries, or the presence of phase impurity. Exposing **1‐TCM^SC^** to vapors of MeOH, DCM, NB, ClBz, and DCB over periods ranging from 24 hours to 2 weeks led to amorphous or low‐crystalline samples, indicating that these solvents damage the SC and disrupt the framework. Interestingly, exposure to EtOH vapors led to a SC‐to‐SC transformation yielding **1‐H_2_O^SC^** (whose structure was described already above).^[^ ^18^ ^]^ Notably, this new phase did not revert to the initial SC phase even after 2 weeks of exposure to TCM vapors, denoting the transformation irreversibility. Single‐crystalline‐to‐powder (SC‐to‐Pwd) transformation resulted instead from the exposure of **1‐TCM^SC^** to MeCN vapors after 24 hours. Comparison between the P‐XRD pattern of this species with that simulated derived from **1‐MeCN^SC^** indicates a good match (see Figure 3) and allows to identify the new microcrystalline powder as **1‐MeCN^Pwd^**.

**Figure 4:**
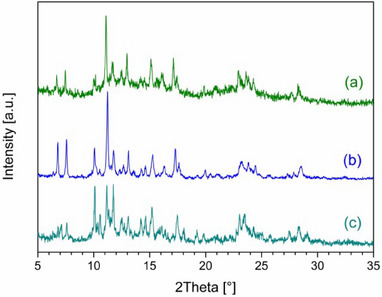
Comparison of experimental P‐XRD data of the powder obtained after 2‐week exposure of **1‐Amorph^Pwd^** to aromatic solvents: DCB (a), ClBz (b) and NB (c).

The underlying structural change that occurs and that can be easily appraised by looking at the SC data relative to the two different CPs metal‐ligand‐solvent organizations is quite striking. It must be reminded here that no apparent dissolution and re‐crystallization of the sample is occurring. Therefore, the process involves the transformation of the 1D ribbon chains into a 2D framework (virtual voids decrease from 35.8% to 13.7%).

In a similar way, a SC‐to‐Pwd process is also triggered by exposure to MeNO_2_ and the corresponding P‐XRD pattern is very similar to that of **1‐MeCN^Pwd^** (Figure 3).

On the basis of what observed so far on the SC and microcrystalline CPs obtained, we can speculate that when the solvent is characterized by the ability to act as H‐bond donor (TCM and H_2_O) its replacement within the crystal lattice proceeds through a rearrangement of the packing but does not involve any change in the CP dimensionality as well as in the status of the uncoordinated chloride ion; by contrast, in the case of MeCN and MeNO_2_ which are significantly poorer H‐bond donors (TCM α = 2.2; MeCN α = 1.5; MeNO_2_ = 1.8; water α = 2.819),^[^ ^19^ ^]^ the rearrangement of the crystal structure is by far more significant (1D to 2D dimensionality change along a change in the coordination geometry from square pyramidal to octahedral). The versatility of the copper(II) ion in terms of its coordination geometries preference surely has an important role in the CP behavior.

Intrigued by these findings, we have also tried to obtain **1‐TCM^SC^** as a microcrystalline powder (SI), an essential step to bridge the gap between interesting observations and practical applications. Despite several attempts, the fast synthesis procedure yielded only an amorphous phase, named **1‐Amorph^Pwd^** (Figure S11a). Although this phase, as‐synthesized, is shown to contain still a certain amount of guest solvent TCM and a little water (Figure S11b), we assume that it is still a CP composed of linear arrays, although disordered. Interestingly, exposure of **1‐Amorph^Pwd^** to TCM vapors for 2 weeks led to a microcrystalline state, which can be identified as a phase compatible with that of **1‐TCM^SC^** and thus referred to as **1‐TCM^Pwd^** (see Figure S12). P‐XRD data comparison with the simulated pattern from **1‐TCM^SC^** shows a fair match, although not perfect. TGA‐FTIR analysis also confirms the presence of TCM, but in amounts approx. 25% lower than expected from SC data (Figure S13). We believe this situation could be due to a slow kinetic of adsorption in this particular case. Similarly, but with a considerably faster rate, exposure to MeCN vapors led to a transformation, within just 2 hours, into a crystalline phase, named **1‐MeCN^Pwd^** as it corresponds to the 2D CP **1‐MeCN^SC^** (see Figures 3 and S14a). TGA‐FTIR also confirms the presence of MeCN in amounts of ca. 2 molecules per metal ion in the CP (Figure S14b). This process was previously observed in a SC‐to‐Pwd fashion starting from **1‐TCM^SC^**. Both **1‐TCM^Pwd^** and **1‐MeCN^Pwd^** phases are stable in open air. As far as the reversibility of the solvent exchange is concerned, **1‐MeCN^Pwd^** exposed to MeOH or EtOH, NB, ClBz, and DCB showed no sign of transformation, while in the presence of TCM, we observed a partial transformation but, at the same time, over longer periods, the sample becomes amorphous, precluding the reversibility of the process. Exposure of **1‐Amorph^Pwd^** to MeNO_2_, a solvent structurally related to MeCN, affords microcrystalline materials whose P‐XRD pattern is very similar to that of **1‐MeCN^Pwd^** (Figure 3). A similar behavior was previously observed in a SC‐to‐Pwd process. TGA‐FTIR analysis, however, confirms the presence of the adsorbed solvent in quantities compatible to ca. 2 molecules of solvent per metal ion in the CP (Figure S15). In the presence of MeOH or DCM vapors, **1‐Amorph^Pwd^** transforms into stable microcrystalline phases, still structurally unidentified (Figures S16a and S16b). A different behavior was observed with EtOH, as a transition into a low‐crystalline phase occurred, whereupon, in contact with air, it became completely amorphous after 20 minutes. Considering their role in atmospheric pollution, we also tested aromatic VOCs as potential targets for **1‐Amorph^Pwd^**. Exposure to NB, ClBz, and DCB led to different results. Interestingly, a clear transformation to microcrystalline phases is evident although comparison among the P‐XRD patterns recorded after exposure fails to discriminate between the species (Figure 4). This notwithstanding, different degrees of crystallinity are observed, with the sample exposed to NB that can be considered the worst in this respect, and the one with ClBz the best. Therefore, TGA/FTIR was performed on all samples in order to gather additional information on the adsorption process. Data showed that, in all cases, the VOC solvents can be clearly identified by FT‐IR in the desolvation stream (Figures S17‐S19). In samples exposed to DCB and NB, Hi‐res TGA profiles identify multi‐step desolvation processes. For example, in the sample exposed to NB, at least three different desolvation events can be detected (Figure S17), characterized by decreasing mass loss percentages along T increase (about 9%, 6%, and 3%, with the maximum desolvation rate at 70 °C, 99 °C, and 113 °C, respectively) and an estimated total adsorbed NB quantity of ca. 2 molecules per metal ion in the CP. Also, in the DCB case, ca. 2 molecules of VOC per metal ion can be estimated to be adsorbed in the CP, with the Hi‐res TGA profiles showing two distinct desolvation processes (about 8.5% with the maximum desolvation rate at 73 °C and 6% at 99 °C as seen in Figure S18). As to the CP exposed to ClBz, a monotonous mass decrease is observed, with no easily discernible change in curve slope and thus no clear separated desolvation steps (Figure S19). This notwithstanding, again ca. 2 molecules of ClBz are shown to be released by increasing T (15% mass loss). In general, 2 molecules per metal unit correspond to ca. 2 mmol of adsorbate per gram of adsorbent material.

**Figure 5:**
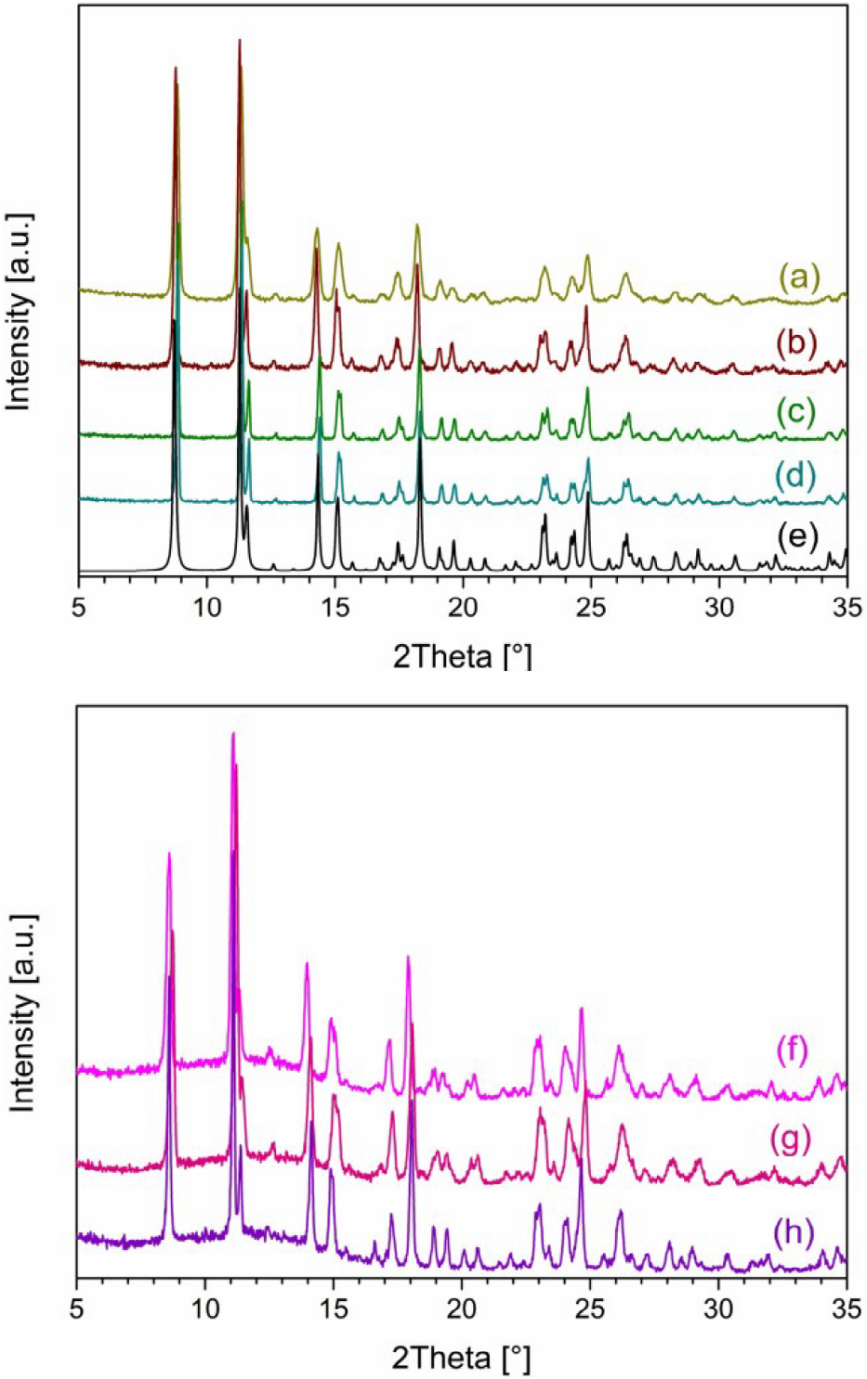
Selective adsorption experiments conducted on **1‐Amorph^Pwd^** over 2 weeks, demonstrating a preferential affinity for MeCN solvent. Comparison between the experimental P‐XRD patterns of **1‐Amorph^Pwd^** exposed to vapors of MeCN and aliphatic solvents (above): MeOH (a), MeNO_2_ (b), DCM (c) and TCM (d), with the simulated P‐XRD of **1‐MeCN^Pwd^** (e). Experimental P‐XRD patterns of **1‐Amorph^Pwd^** exposed to vapors of MeCN and aromatic solvents (below): NB (f), ClBz (g) and DCB (h).

Finally, we embarked on a series of selectivity tests by exposing **1‐Amorph^Pwd^** to a number of binary vapor mixtures of MeCN/TCM, MeCN/MeOH, MeCN/DCM, and MeCN/MeNO_2_ for 2–21 days, and MeCN/NB, MeCN/ClBz, and MeCN/DCB. Results show that MeCN is always preferentially adsorbed, as the formation of **1‐MeCN^Pwd^** is observed by P‐XRD analysis (see Figure 5). This result is quite interesting as it reveals quite a marked selectivity. Interestingly, in the case of MeCN/TCM mixture, we monitored the process over 2 weeks, in order to ascertain **1‐MeCN^Pwd^** to be the preferential product regardless of the slower kinetic of adsorption seen with TCM. As to the case of the MeCN/MeNO_2_ mixture, TGA/FTIR was employed due to the marked resemblance of the P‐XRD patterns of the two resulting species, and the analysis demonstrated that MeNO_2_ is preferentially adsorbed (Figure S20), thus leading to **1‐MeNO_2_^Pwd^**. Thus, we can determine that **1‐Amorph^Pwd^** is characterized by an adsorption selectivity in the following order: MeNO_2_ > MeCN > all others, and an amount of adsorbate of ca. 2 mmol/g. For comparison's sake, HKUST‐1 was reported to be able to adsorb, after activation, about 6 mmol/g of MeNO_2_, while its Fe(II) analogue, ca. 2 mmol/g, as described in recent reports, which however do not provide information about selectivity.^[^ ^20^ ^]^

### 2.6 Variable Temperature Powder X‐ray Diffraction (VT P‐XRD)

In situ VT P‐XRD experiments were performed on both **1‐TCM^Pwd^** and **1‐MeCN^Pwd^**. They showed good stability for both CP samples up to 383 K, consistent with SC‐XRD data. The powder patterns in Figures S21,S22 (Supporting Information) display the characteristic peaks of the crystalline phases identified from SC‐XRD. In the case of **1‐TCM^Pwd^**, shown in Figure S21, the intensity of the highest peak positioned at 2*θ* = 11.72° diminished, *I*_max_ (303 K) = 1.6 × *I*_max_ (383 K), with a slight increase of an amorphous fraction that becomes more relevant at 423 K. Finally, at 443 K, the **1‐TCM^Pwd^** sample began to melt, causing the diffracted peaks to disappear.

Sample **1‐MeCN^Pwd^** maintained good crystallinity up to 383 K (see Figure S22). As the temperature increased to 423 K, there was a notable decrease in the intensity of the characteristic peaks of **1‐MeCN^Pwd^**, accompanied by the emergence of new diffracted peaks. This indicates the coexistence of **1‐MeCN^Pwd^** with a newly formed phase. At 443 K, the just‐mentioned phase transition becomes even more evident.

Overall, these data confirm the thermal stability and structural integrity of both CPs up to ca. 400 K, and align with the VT SC‐XRD data and with the hot‐stage microscopy analysis. In both cases, solvent molecules (TCM and MeCN) are trapped within isolated pockets. In the case of TCM, numerous solvent‐chain interactions are detected, clearly limiting the ease of solvent escape under heating. In **1‐MeCN^SC^**, where the MeCN molecules are less interacting with the framework, stronger interchain interactions are present, as described above, and by providing greater resistance to structural reorganization, they are probably responsible for the stability of this CP under comparable conditions. These findings, from VT SC‐XRD measurements and SC‐to‐SC transformation, further emphasize the fundamental differences between thermal and chemical stimuli when investigating dynamic systems such as those described here. Specifically, it is important to recognize that the tested CPs exhibit thermal stability up to 400 K while simultaneously do exchange solvent at room temperature. This apparent contradiction arises because thermal stimuli primarily influence bulk structural integrity and desolvation kinetics, whereas chemical stimuli rely on localized, reversible interactions, involving both exchanging guests, that can occur under much milder conditions and by a different mechanism.

## 3 Conclusion

This study provides an in‐depth exploration of novel bispidine‐based Cu(II) CPs with a particular emphasis on their structural features, adsorption and solvent exchange capabilities. From a structural point of view, as demonstrated by structural characterization of SCs, but also by the solvent exchange processes observed both in a SC‐to‐SC and SC‐to‐Pwd fashion, bispidine **L1** forms with Cu(II) ions preferentially either 1D or 2D CPs depending on the H‐bonding donor capability of the adsorbed solvent, and in particular, on its ability to interact with chloride anion. Indeed, **1‐TCM^SC^** and **1‐H_2_O^SC^** (studied by SC‐XRD analysis) are 1D CPs with ribbon‐like topology, and they are characterized by a pentavalent Cu(II) ion which is coordinated to only one chloride ion, while the other anion is involved in extensive HB interaction with the solvent. On the contrary, **1‐MeCN^SC^**, obtained as SC, and the species **1‐MeNO_2_^Pwd^** characterized only as microcrystalline powder, are 2D CPs with a topology never observed before in bispidine‐based CPs, and they exhibited a more stable extended network with reduced void spaces. Remarkably, when **1‐TCM^SC^** is exposed to MeCN vapors it transforms to **1‐MeCN^Pwd^** via a SC‐to‐Pwd transformation which entails a massive reorganization of the entire CP, especially in terms of metal‐ligand connectivity. Based on the available structural data, this phenomenon can be attributed to the high adaptability of the Cu(II) ion, which supports various coordination geometries, and the network of hydrogen‐bonding interactions facilitated by a suitable solvent. If confirmed through broader future investigations, this intriguing aspect, previously unnoticed in bispidine‐based CPs, could offer a new pathway for achieving dynamic behavior in solid‐state materials. VT SC‐XRD experiments were also conducted on **1‐TCM^SC^** and **1‐MeCN^SC^**, revealing a low tendency for both CPs to release solvent molecules by a thermal stimulus, thereby highlighting their significant thermal stability. This behavior contrasts with the findings from TGA experiments, which show solvent‐loss at temperatures comparable to the boiling point of the trapped solvent, a discrepancy that could be related to the different experimental conditions employed in each technique.

Attempts to reproduce **1‐TCM^SC^** as microcrystalline powders led to a desolvated amorphous phase **1‐Amorph^Pwd^** which therefore was subjected to extensive adsorption experiments by exposing it to single solvents or solvents binary mixtures at *r.t*. and monitored by both P‐XRD and TGA‐FT‐IR analysis. Main results show a remarkable ability to quickly adsorb MeCN and MeNO_2_, with a marked preference for the latter when the two were put in competition; not only that, **1‐Amorph^Pwd^** was shown to be able to trap aromatic VOCs up to amounts of ca. 2 solvent molecules per metal, which corresponds to about 2 mmol/g. These adsorption capabilities are especially significant because they more closely replicate real‐world application conditions than other studies reported in the literature. Specifically, the experimental conditions used respond to the need of a powdered adsorbent capturing VOCs at room temperature directly from a polluted atmosphere. Furthermore, the data already account for N₂ and O₂ competition, as the experiments were conducted at *r.t*. and 1 atm pressure, with no prior activation of the adsorbent. More generally, these findings open new avenues for designing dynamic, selective adsorbents for environmental and industrial applications. Future research will explore additional structural diversification and, ultimately, the scaling up of these materials for practical usage and investigating their performance under more diverse competitive conditions.

## 4 Experimental Section

### Materials

The bispidine ligand **L1** (C_23_H_26_N_4_O_5_, Dimethyl‐3,7‐dimethyl‐9‐oxo‐2,4‐di(pyridin‐4‐yl)‐3,7‐diazabicyclo‐[3.3.1]nonane‐1,5‐dicarboxylate) (see Scheme 1) was synthesized following the previously reported procedure.^[^ ^6g^ ^]^ Copper chloride dihydrate (CuCl_2_·2H_2_O, ≥99.0%) and the solvents: acetonitrile (MeCN, 99.8%), methanol (MeOH, 99.85%), ethanol (EtOH, 99.5%), nitromethane (MeNO_2_, 99.0%), dichloromethane (DCM, 99.5%), trichloromethane (TCM, 99.8%), chlorobenzene (ClBz, 99.8%), 1,2‐dichlorobenzene (DCB, 99.0%), nitrobenzene (NB, 99.0%), and tetrahydrofuran (THF, 99.9%) were commercially available and used as received.

### Synthesis

The bispidine‐based Cu(II) CPs have been prepared in mono‐ and micro‐crystalline forms by reacting CuCl_2_·2H_2_O with **L1** under various conditions. Details on the synthetic procedures are given in Supporting Information.

### Single crystal X‐ray diffraction (SC‐XRD)

SC‐XRD data were collected on a D8 VENTURE (Bruker AXS) diffractometer operating with an Incoatec microfocus source, generating monochromatic radiation and a Photon III CPAD detector. Intensity data were collected using the Mo *K*α radiation (λ = 0.71073 Å) for **1‐TCM^SC^** and **1‐H_2_O^SC^**. While for **1‐MeCN^SC^** the Cu *K*α radiation (λ = 1.54178 Å), due to the smaller dimensions. The temperature of SC was maintained at 100(2) K during the measurement using an Oxford Cryostream 800 plus flow nitrogen cryostat. The measurement strategy consisted of *ω* and *ϕ* scans with a frame width of 1.0°. The data collection strategy, along with the data integration and the multi‐scan absorption correction, were executed using the APEX5 v.2023 suite^[^ ^21^ ^]^ incorporating SAINT 8.40a and SADABS‐2016/2 tools.^[^ ^22^, ^23^ ^]^ The collected intensity data yield unique reflections merged in the 2/*m* Laue class for all studied CPs (0.051 ≤ *R*_int_ ≤ 0.089). The intensity statistics, based on normalized structure factors, 0.903 ≤|E^2^−1|≤ 0.948, suggested centrosymmetric space groups. The systematic absences pointed to the *P*2_1_ */c*, *C*2*/c*, and *P*2_1_ */n* space groups for **1‐TCM^SC^**, **1‐H_2_O^SC^**, and **1‐MeCN^SC^**, respectively, guiding the structural determinations accordingly. The structure solution was carried out by direct methods with ShelXT v.2018/2,^[^ ^24^ ^]^ and refinement by full‐matrix least‐squares based on *F* ^2^ with ShelXL v.2018/3,^[^ ^25^ ^]^ all implemented within the Olex 2 v.1.5 program.^[^ ^26^ ^]^ After performing the solvent‐exchange experiments, the retrieved SC samples were analyzed using SC‐XRD method to identify any structural transformations, whether involving a SC‐to‐SC or a SC‐to‐Pwd transition. For the latter, P‐XRD patterns were acquired within the 2*θ* range from 4 to 35° using Cu *K*_α_ radiation, in a full *ϕ*‐scan (*ϕ* = 360°) mode, with the detector positioned at 80 mm from the sample.

Deposition Number(s) 2427190 (for **1‐TCM^SC^**), 2 427 193 (for **1‐H_2_O^SC^**), 2 427 192 (for **1‐MeCN^SC^**) contain the supplementary crystallographic data for this paper. These data are provided free of charge by the joint Cambridge Crystallographic Data Centre and Fachinformationszentrum Karlsruhe http://www.ccdc.cam.ac.uk/structures Access Structures service.

### Variable‐temperature single crystal X‐ray diffraction (VT SC‐XRD)

VT SC‐XRD measurements were performed on suitable SCs of **1‐TCM^SC^**, glued on a glass fiber, and **1‐MeCN^SC^** glued on a Cryo‐Loop. Intensity data were collected using monochromatic Mo *K*_α_ radiation (*λ* = 0.71073 Å) for **1‐TCM^SC^** and monochromatic Cu *K*_α_ radiation (*λ* = 1.54178 Å) for **1‐MeCN^SC^**, across a temperature range from 100 K to 400 K, by using different temperature increments (10‐50 K). During the measurements, a steady nitrogen stream regulated by an Oxford Cryostream system was directed over the SC samples to maintain temperature stability. The unit‐cell parameters were determined at each temperature using the least‐squares method from a set of more than 1500 reflections within the 2*θ* range 4‐60°. The collected intensity data were processed using the structural model from the 100 K dataset.

### In‐silico analysis

The crystal packings of **1‐TCM^SC^** and **1‐MeCN^SC^** were analyzed with CCDC Mercury (v. 2024.1.0).^[^ ^15^ ^]^ To further investigate the intermolecular interactions which trap the solvent molecules in the solid forms, Crystal‐Explorer 21.3 was used.^[^ ^27^ ^]^ The HSs and their associated 2D FPPs were computed, by using the structure's crystallographic information file (CIF) as input. Given that in **1‐TCM^SC^** one solvent molecule is disordered over two positions, HSs and FPPs were computed by using the most representative model in terms of occupancy factors (0.866 vs. 0.134).

### Hot‐stage microscopy

Selected SC of the studied Cu(II)‐based bispidine CPs were placed in a closed top stage (Linkam 600 Heating/Freezing Stage, Linkam Scientific Instruments, Surrey, United Kingdom) using a 50× objective (Nikon Microscope Eclipse 80i, Nikon Instruments, Melville, NY, USA), heated at 5 K/min to 453 K, via a remote temperature controller (Linkam TP94, Linkam Scientific Instruments, Surrey, United Kingdom). Measurements were carried out under a dry N_2_ atmosphere. An online camera (Nikon Digital Still Camera DXM1200 for Microscope, Nikon Instruments, Melville, NY, USA) recorded the sample for each temperature step under the microscope. After the heating cycle, the sample was cooled down to *r*.*t*. at the same rate as the heating.

### P‐XRD

Powder diffraction data were collected using a Bruker New D8 Da Vinci diffractometer employing the Cu *K*_α1_ radiation = 1.54056 Å, equipped with a Bruker LYNXEYE‐XE detector, an Anton Paar XRDynamic 500 diffractometer equipped with the Co *K*_α1_ radiation = 1.78897 Å, and a Pixos 2000 CdTe detector. The P‐XRD data were recorded, in Bragg/Brentano geometry, at *r*.*t*. on finely ground samples dispersed on a Si‐zero‐background sample holder. The scanning covered the angular range of 2*θ* (°) from 5 to 50, with a step size of 0.02° and an exposure time of 0.5 s/step.

### VT P‐XRD

VT P‐XRD measurements were performed using an Anton Paar XRDynamic 500 diffractometer in Bragg‐Brentano geometry. The **1‐TCM^Pwd^** and **1‐MeCN^Pwd^** powder samples were ground and placed onto the mid‐temperature chamber sample stage of an Anton Paar TTK600. P‐XRD data were collected while heating in air from 303 to 423 K in 40 K intervals, and from 423 to 443 K (and up to 463 K for **1‐MeCN^Pwd^**) at 20 K intervals, at a heating rate of 10 K/minute. The diffraction patterns were recorded in the 2*θ* range of 5–50° with a step size of 0.02° and an exposure time of 80 seconds per step.

### Thermogravimetry (TG)

TG coupled with FT‐IR analysis of the gases evolved was used to evaluate the amount of VOC desorbed and to identify it. The measurements were performed using a Thermobalance Q5000IR (TA Instruments) connected through a transfer line to a FT‐IR Agilent Technologies spectrophotometer (Cary 640). Around 15 mg of samples were heated in Pt crucibles under nitrogen flow as the purging gas. More in detail, an initial TG analysis of **1‐Amorph^Pwd^** powder sample was performed at 10 °C·minute^−1^ heating rate between 25 °C and 250 °C to assess the temperature range in which it remains stable. Also, preliminary studies showed that the exposure to various solvents did not alter the degradation onset temperature of CPs and that the desorption and degradation signals were well separated. Starting from these findings, the other measurements were then performed in the range 25–150 °C and, since the desorption gave rise to rather broad and continuous mass loss signals, the dynamic Hi‐Res mode was chosen in order to enhance the separation of the different mass losses. In this mode, allowed by the instrument software (TA Universal Analysis 2000), the heating rate decreases as the derivative of the mass loss (dmass%/dT) increases and vice versa. In the experiments performed, the heating rates ranged from a minimum of 2 °C·minute^−1^ to a maximum of 20 °C·minute^−1^, setting the resolution to 4.0 and the sensitivity to 1. The gases evolved from TGA were transferred to the FT‐IR spectrometer with a nitrogen flow of 70 mL·minute^−1^. FT‐IR spectra were acquired every 30 seconds in the range 400–4000 cm^−1^ with a 4 cm^−1^ width slit. The optical bench was purged with nitrogen (N_2_) and a background spectrum was recorded before each analysis. The measurements were repeated three times on representative samples and the mass losses are reported as mean value with an error of ± 1%. The identification of the FTIR spectra was performed using the Vapor Phase (Gas) Infrared Spectra of Spectrabase repository (free version) of John Wiley & Sons, Inc.

### Solvent adsorption, selective adsorption, and exchange experiments

The experiments began with solid‐vapor adsorption tests. The amorphous powder sample, **1‐Amorph^Pwd^**, obtained from the fast crystallization procedure (see details in Supporting Information, Section S1.2), was exposed to various solvent vapors, including MeOH, EtOH, MeCN, MeNO_2_, DCM, TCM, NB, ClBz, and DCB for durations ranging from 1 hour to 2 weeks, allowing thus to monitor the effects of solvent exposure over time. The experimental setup involved placing the sample in a sealed chamber alongside a vial containing the volatile solvent to maximize solid‐vapor interaction. In parallel, solvent‐exchange experiments were conducted using a similar setup on SC and microcrystalline phases of **1‐TCM^SC^** and **1‐MeCN^SC^** with the same solvents for the same duration as the adsorption experiments. After each specified exposure period, the samples were retrieved and immediately analyzed using X‐ray diffraction (XRD) to detect structural transformations. Experiments were also conducted in which the amorphous powder sample was simultaneously exposed to vapors of two distinct solvents to evaluate the selectivity in adsorbing specific solvent(s). Similarly, the selective solvent‐exchange behavior was assessed in powder samples of the studied CPs. Table S8 provides a summary of the procedures followed to obtain all CP phases described in this work.

## Supporting Information

The authors have cited additional references within the Supporting Information.^[^ ^12^, ^13^, ^15^ ^]^

## Conflict of Interests

The authors declare no conflict of interest.

## Supporting information



Supporting Information

## Data Availability

The data that support the findings of this study are available from the corresponding author upon reasonable request.
