# Supplementary material for: Bispidine‐Based Copper(II) Coordination Polymers with Remarkable Dynamic Properties, Selective Volatile Organic Compounds Adsorption, and Exchange Capabilities
Source: Chemistry. 2025 May 27;31(36):e202501431. doi: 10.1002/chem.202501431 (PMC12202842; doi:10.1002/chem.202501431)
Supplement: Supplementary file 1 — Supporting Information [file CHEM-31-e202501431-s001.docx]

**Supporting Information**

**Bispidine-Based Copper(II) Coordination Polymers with Remarkable Dynamic Properties, Selective Volatile Organic Compounds Adsorption and Exchange Capabilities**

Meriem Goudjil,^a^ Martina Lippi ^* a^ , Chiara Pelosi,^b^ Luca Bernazzani,^b,c^ Patrizia Rossi,^a^ Paola Paoli,^a^ and Massimo Cametti ^* d^

^1^ Department of Industrial Engineering, University of Florence, Via S. Marta 3, Florence 50139 (Italy)

^2^ Department of Chemistry, Materials and Chemical Engineering “Giulio Natta”, Politecnico di Milano, Via Luigi Mancinelli, 7, Milano 20131 (Italy)

^3^ Dipartimento di Chimica e Chimica Industriale, Università di Pisa, Via Moruzzi 13, 56124, Pisa (Italy)

^4^ Institute for the Chemistry of OrganoMetallic Compounds-ICCOM, Italian National Research Council-CNR, via G. Moruzzi 1, 56124, Pisa, (Italy)

**TABLE OF CONTENT**

1. Synthesis p. S2

*1.1 Preparation of single crystals (SCs)* p. S2 - S3

*1.2 Preparation of powder (Pwd) samples* p. S3

2. Single crystal X-ray characterization (SC-XRD) p. S4

*2.1 Single crystal X-ray diffraction analysis (SC-XRD)* p. S4 - S6

*2.2 Crystal Structure Description* p. S7 - S16

3. Hirshfeld surface analysis p. S17

4. Variable temperature single crystal X-ray diffraction (VT SC-XRD) p. S18

5. Hot-stage microscopy p. S19

6. Powder X-ray diffraction (P-XRD) and thermogravimetry (TG)

coupled with FTIR spectroscopy of evolved gases p. S20-S29

7. Variable temperature powder X-ray diffraction (VT P-XRD) p. S30

8. Summary of the procedure for obtaining Cu-based coordination polymers p. S31

- - - 1. **Synthesis**

*1.1 Preparation of single crystals (SCs)*

The crystallization of high-quality SCs of new Cu-bispidine coordination polymers has been successfully achieved by applying two crystallization procedures. The first method involved a liquid-liquid slow diffusion process using three miscible solvents, creating “a three-layer system”. This process began with introducing a trichloromethane (TCM) solution of ligand **L1** at the base of the crystallization vessel. A less dense solvent was then carefully added as an intermediate layer to facilitate controlled crystal growth. Finally, a methanol (MeOH) solution containing CuCl_2_·2H_2_O was gently layered on top.

Second, the slow evaporation method involved carefully mixing a solution containing the ligand dissolved in TCM with a solution of CuCl_2_·2H_2_O dissolved in MeOH or MeCN solvents.

The preparation procedure for each CP is given in detail hereafter.

**Crystals of 1-TCM^SC^**, {Cu(C_23_H_26_N_4_O_5_)_2_Cl_2_·4(CHCl_3_)}_n_

25 mg (0.057 mmol) of **L1** were dissolved in 1.5 mL of TCM. Over this solution, 2.5 mL of THF was meticulously added drop by drop, ensuring a controlled addition of a top layer. Subsequently, a solution of 4.94 mg (0.029 mmol) of CuCl_2_·2H_2_O in 1 mL of MeOH was introduced, completing the third and final layer. After one day, dark-blue prismatic crystals precipitated at the bottom of the crystallization tube and grew up to 500 μm in length over the next few days.

**Crystals of 1-MeCN^SC^**, {Cu(C_23_H_26_N_4_O_5_)_2_Cl_2_·2(CH_3_CN)}_n_

Following the three-layer method, 25 mg (0.057 mmol) of **L1** were dissolved in 1.5 mL of TCM and loaded in the crystallization tube. A 2.5 mL layer of THF followed this, and finally, a solution of 4.94 mg (0.029 mmol) of CuCl_2_·2H_2_O in 1 mL of MeCN was carefully introduced, completing the third layer. Within five days, green tabular crystals (measuring up to 100 μm in length) formed at the bottom of the crystallization tube.

Using the slow-evaporation method, 150 mg (0.057 mmol) of **L1** were dissolved in 5 mL of TCM. To this solution, 29.64 mg (0.029 mmol) of CuCl_2_·2H_2_O dissolved in 3 mL of MeCN were slowly added, and the mixture was stirred for an hour, resulting in a limpid green solution. Overnight, blue prismatic crystals formed at the bottom of the crystallization vessel. These crystals were identified as the **1-TCM^SC^** phase based on SC-XRD analysis. On moving the vessel, they started turning green. However, keeping the vessel immobile, crystals turned green within two days. SC-XRD analysis indicated that they belong to the **1-MeCN^SC^** phase.

*1.2 Preparation of powder (Pwd) samples*

The specific procedures and conditions for obtaining Pwd samples are detailed below.

**Microcrystalline powder of 1-TCM^Pwd^**

While stirring, a MeOH solution of CuCl_2_·2H_2_O (3 mL, 29.64 mg) was gradually added to a TCM solution of **L1** (5 mL; 150 mg). A green solution initially formed and progressively turned blue after 5 minutes of stirring, accompanied by a dark blue precipitate. The solid, filtered and dried in a desiccator, is light blue.

The fast crystallization method resulted in an amorphous powder. By exposing this sample to vapors of TCM for two weeks, a microcrystalline powder of **1-TCM^Pwd^** was successfully formed, as confirmed by P-XRD data (see Section S6, Figure S12).

**Microcrystalline powder of 1-MeCN^Pwd^**

The microcrystalline powder of **1-MeCN** can be obtained by directly exposing the above-mentioned amorphous powder to MeCN vapors for just two hours (Fig.S14a), this occurs through an adsorption process. The **1-MeCN^Pwd^** can also be obtained via a solvent-exchange mechanism by subjecting the microcrystalline powder of **1-TCM^Pwd^** to MeCN vapors for the same duration. Furthermore, it forms through simultaneous exposure of the amorphous Pwd sample to MeCN vapors in the presence of other solvents over a period of two weeks. More details can be found in the Results and Discussion section.

- - - 1. **Single crystal X-ray characterization (SC-XRD)**

*2.1. Single crystal X-ray diffraction analysis (SC-XRD)*

In **1-TCM^SC^**, the asymmetric unit contains one copper ion, two chloride ions, two crystallographically independent **L1** molecules, and four TCM solvent molecules, leading the formula moiety {[Cu(C_23_H_26_N_4_O_5_)_2_Cl]Cl·4(CHCl_3_)}. All non-hydrogen atoms were refined anisotropically. Hydrogen atoms were geometrically positioned, and their thermal parameters were refined using the riding model (1.2 × *U*_eq_ for aromatic C—H and 1.5 × *U*_eq_ for those binding to methyl groups). One TCM molecule exhibiting positional disorder was split over two sites and refined using PART1/PART2 and FVAR constraints, with site occupancy factors (s.o.f.) of 0.866/0.134(2). DFIX (1.76±0.01), DANG (2.83±0.01), and EADP constraints were employed to model the disorder. Finally, the refinement was concluded by omitting two reflections with outliers. The model with the most representative TCM molecule, exhibiting the higher s.o.f. of 0.866(2), was considered for the structural description.

In **1-H_2_O^SC^**, the asymmetric unit comprises one copper ion, two chloride ions, two crystallographically independent **L1** ligand molecules (referred to as **L1A** and **L1B**), similar to the asymmetric unit of **1-TCM^SC^**. Additionally, there are seven co-crystallized water (H_2_O) molecules.

Each ligand, **L1A** and **L1B**, shows positional disorder in one of its ester groups, which has been refined across two sites (designated as **L1A** and **L1C**; and **L1B** and **L1D**). The refinement process involved using PART1/PART2 instruction with the s.o.f. fixed to 0.50 for both disordered sites, in addition to DFIX constraints to model this disorder (1.205±0.001 Å to C=O, 1.315±0.005 Å to C—O and 1.445±0.005 Å to O—CH_3_).

Regarding H_2_O molecules, four are fully occupied, while the remaining three are partially filled, with s.o.f. fixed to 0.25 for O5W and O6W and to 0.15 for O7W, yielding a total of 4.65 H_2_O molecules per formula unit. Consequently, the chemical formula of the hydrated CP is {Cu(C_23_H_26_N_4_O_5_)_2_Cl_2_·4.65(H_2_O)}.

Non-hydrogen atoms were refined anisotropically, except for disordered atoms and partially occupied H_2_O molecules. H-atoms binding to the ligand molecules were placed at calculated positions. The H-atoms of fully occupied H_2_O molecules were found in the difference Fourier map; their geometry was modeled introducing DFIX (0.84±0.01 Å) and DANG (1.33±0.02 Å) constraints. H-atoms of partially occupied water molecules could not be resolved and have not been included in the graphical illustrations. Finally, the isotropic thermal parameters of H-atoms were refined using the riding model according to the atoms to which they are bonded.

The structural packing of **1-H_2_O^SC^**, described in the “Results and discussion” section, is examined through two models: the first model includes **L1A** and **L1B**; while the second involves **L1C** and **L1D**. In both models, only fully occupied co-crystallized H_2_O molecules were considered.

In **1-MeCN^SC^**, the asymmetric unit includes half of a CuCl_2_ moiety, one **L1** ligand, and one MeCN solvent molecule corresponding to the formula moiety {Cu(C_23_H_26_N_4_O_5_)_2_Cl_2_·2(CH_3_CN)}.

Non-hydrogen atoms were refined anisotropically. H-atoms binding to the ligand were all fixed at calculated positions, and their thermal parameters were refined using the riding model. For the MeCN solvent molecule, H-atoms were found in the difference Fourier map and refined using the riding model. The methyl C—H interatomic distances of MeCN were refined using DFIX (0.97±0.01 Å) and DANG (1.567±0.01 Å) constraints.

Table S1 summarizes details of the data collection conditions and selected refinement parameters. The crystallographic projections were generated using Mercury v.4.0.^^[[1]](#footnote-1)^^

**Table S1.** Crystal data and structure refinement parameters for Cu-Bispidine coordination polymers.

| **Structural parameters** | **1-TCM^SC^ (CDCD code:2427190)** | **1-H_2_O^SC^ (CDCD code:2427193)** | **1-MeCN^SC^ (CDCD code:2427192)** |
| --- | --- | --- | --- |
| empirical formula | (C_46_H_52_Cl_2_CuN_8_O_10_)·4(CHCl_3_) | (C_46_H_52_Cl_2_CuN_8_O_10_)·4.65(H_2_O) | (C_46_H_52_Cl_2_CuN_8_O_10_)·2(CH_3_CN) |
| formula weight (g·mol^-1^) | 1488.86 | 1095.17 | 1093.50 |
| temperature (K) | 100(2) | 100(2) | 100(2) |
| crystal system | monoclinic | monoclinic | monoclinic |
| space group | *P*2_1_/*c* | *C*2/*c* | *P*2_1_/*n* |
| *a* (Å) | 10.3655(16) | 31.193(5) | 10.6588(8) |
| *b* (Å) | 23.067(4) | 23.069(4) | 11.7433(9) |
| *c* (Å) | 26.811(5) | 17.124(4) | 20.5441(16) |
| *β* (deg) | 94.829(7) | 120.216(10) | 99.757(3) |
| volume (Å^3^) | 6387.8(18) | 10648(3) | 2534.3(3) |
| *Z* | 4 | 8 | 2 |
| diffractometer | Bruker D8 Venture | Bruker D8 Venture | Bruker D8 Venture |
| ρ_calc_ (mg·m^-3^) | 1.548 | 1.366 | 1.433 |
| absorption correction | multi-scan | multi-scan | multi-scan |
| crystal size (mm) | 0.38 × 0.14 × 0.14 | 0.30 × 0.06 × 0.06 | 0.12 × 0.10 × 0.06 |
| crystal color/shape | blue/prism | blue/prism | green/tabular |
| μ (mm^-1^) | 0.987 | 0.581 | 2.151 |
| *hkl* range | -15 ≤ *h* ≤ 15 | -36 ≤ *h* ≤ 36 | -12 ≤ *h* ≤ 12 |
|  | -34 ≤ *k* ≤ 34 | -26 ≤ *k* ≤ 26 | -13 ≤ *k* ≤ 14 |
|  | -40 ≤ *l* ≤ 40 | -20 ≤ *l* ≤ 20 | -24 ≤ *l* ≤ 24 |
| no. of reflections collected | 275323 | 213054 | 44042 |
| no. of independent reflections | 22934 | 8907 | 4640 |
| *F*(000) | 3036 | 4588 | 1142 |
| *R*_1_ | 0.0576 | 0.0593 | 0.0309 |
| *wR*_2_ | 0.1726 | 0.1564 | 0.0845 |
| GooF | 1.049 | 1.148 | 1.040 |
| transmission factors | *T*_min_ = 0.631, *T*_max_ = 0.746 | *T*_min_ = 0.609, *T*_max_ = 0.745 | *T*_min_ = 0.659, *T*_max_ = 0.753 |
| largest difference map hole | Δρ_min_ = −1.73 e·Å^–3^; Δρ_max_ = 1.66 e·Å^–3^ | Δρ_min_ = −1.28 e·Å^–3^; Δρ_max_ = 1.56 e·Å^–3^ | Δρ_min_ = −0.41 e·Å^–3^; Δρ_max_ = 0.32 e·Å^–3^ |

*2.2. Crystal Structure Description*

*Copper coordination in* ***1-TCM^SC^****,* ***1-H_2_O^SC^*** *and* ***1-MeCN^SC^***

**Table S2.** Geometrical parameters for Cu coordination sphere in **1-TCM^SC^**, **1-H_2_O^SC^** and **1-MeCN^SC^** CPs.

| Coordination Polymer | Bond length (Å) | | Bond angle (°) | |
| --- | --- | --- | --- | --- |
| **1-TCM^SC^** | Cu1—Cl1 | 2.4687 (7) | N1A—Cu1—Cl1 | 95.61(6) |
|  | Cu1—N1A | 2.0364 (20) | N1A—Cu1—N3A^i^ | 165.66(8) |
|  | Cu1—N3A^i^ | 2.0375 (19) | N1A—Cu1—N1B^ii^ | 87.24(8) |
|  | Cu1—N1B^ii^ | 2.0636 (19) | N1A—Cu1—N3B | 91.64(8) |
|  | Cu1—N3B | 2.0575 (19) | N3A^i^—Cu1—Cl1 | 98.33(6) |
|  | Cu1···Cl2 | 4.135(1) | N3A^i^—Cu1—N1B^ii^ | 87.15(8) |
|  |  |  | N3A^i^—Cu1—N3B | 89.35(8) |
|  |  |  | N1B^ii^—Cu1—Cl1 | 99.66(6) |
|  |  |  | N3B—Cu1—Cl1 | 99.49(6) |
|  |  |  | N3B—Cu1—N1B^ii^ | 160.83(8) |
|  | | | | |
| **1-H_2_O^SC^** | Cu1—Cl1 | 2.4565(12) | N1A—Cu1—Cl1 | 100.13(10) |
|  | Cu1—N1A | 2.036(3) | N1A—Cu1—N3A^i^ | 159.53(12) |
|  | Cu1—N3A^i^ | 2.038(4) | N1A—Cu1—N1B^ii^ | 88.47(15) |
|  | Cu1—N1B^ii^ | 2.041(4) | N1A—Cu1—N3B | 89.18(14) |
|  | Cu1—N3B | 2.040(4) | N3A^i^—Cu1—Cl1 | 100.33(8) |
|  | Cu1···Cl2 | 4.051(2) | N3A^i^—Cu1—N1B^ii^ | 90.55(15) |
|  |  |  | N3A^i^—Cu1—N3B | 87.23(15) |
|  |  |  | N1B^ii^—Cu1—Cl1 | 94.50(9) |
|  |  |  | N3B—Cu1—Cl1 | 98.43(10) |
|  |  |  | N3B—Cu1—N1B^ii^ | 167.07(12) |
|  | | | | |
| **1-MeCN^SC^** | Cu1—Cl1 | 2.3442 (4) | Cl1^i^—Cu1—Cl1 | 180.0 |
|  | Cu1—Cl1^i^ | 2.3442 (4) | N1A—Cu1—Cl1^i^ | 90.49(4) |
|  | Cu1—N1A | 2.0272 (13) | N1A^i^—Cu1—Cl1 | 90.49(4) |
|  | Cu1—N1A^i^ | 2.0272 (13) | N1A^i^—Cu1—Cl1^i^ | 89.51(4) |
|  | Cu1—N3A | 2.4896 (12) | N1A—Cu1—Cl1 | 89.51(4) |
|  | Cu1—N3A^iv^ | 2.4896 (12) | N1A—Cu1—N1A^i^ | 180.0 |
|  |  |  | N3A^ii^—Cu1—N3A^iii^ | 180.0 |
|  |  |  | N3A^ii^—Cu1—Cl1 | 89.57(3) |
|  |  |  | N3A^iii^—Cu1—Cl1 | 90.43(3) |
|  |  |  | N3A^ii^—Cu1—Cl1^i^ | 90.43(3) |
|  |  |  | N3A^iii^—Cu1—Cl1^i^ | 89.57(3) |
|  |  |  | N1A—Cu1—N3A^ii^ | 93.35(5) |
|  |  |  | N1A—Cu1—N3A^iii^ | 86.65(5) |
|  |  |  | N3A^ii^—Cu1—N1A^i^ | 86.65(5) |
|  |  |  | N1A^i^—Cu1—N3A^iii^ | 93.35(5) |

Symmetry codes for **1-TCM^SC^**: (i) −*x*+1, *y*+1/2, −*z*+3/2.

Symmetry codes for **1-H_2_O^SC^**: (i) 3/2−*x*, −1/2+*y*, 1/2−*z*; (ii) 3/2−*x*, 1/2+*y*, 1/2−*z*.

Symmetry codes for **1-MeCN^SC^**: (i) −*x*+2, −*y*+2, −*z*+1; (ii) 1/2+*x*, 3/2-*y*, 1/2+*z*; (iii) 3/2-*x*, 1/2+*y*, 1/2−*z*; (iv) 1-*x*, 1-*y*, -*z*.

*Molecular packing*

- **1-TCM^SC^**

In the crystal packing, each independent **L1A** and **L1B** ligand, alternating along the *b*-axis direction, is involved in inter-ribbon interactions with its symmetrically related, via an inversion center, generating H-bonded pairs. These pairs, held together through mutual CH···O contacts involving different sites, as illustrated in Figure S1a.


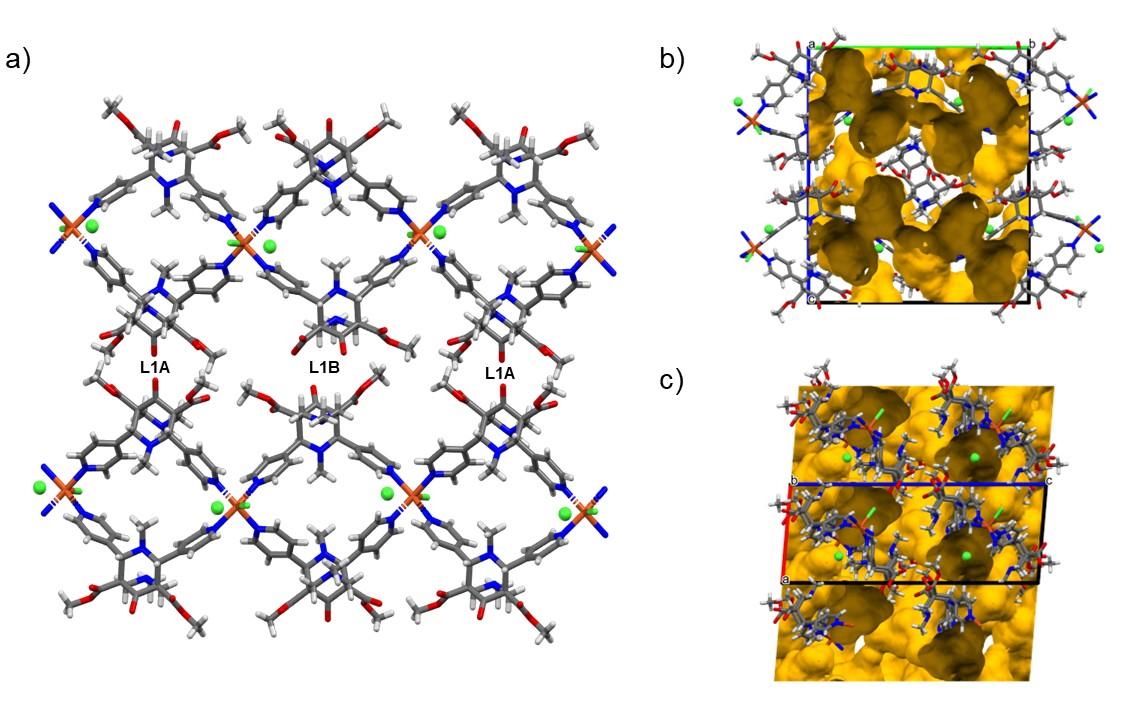


**Figure S1.** Projection of the wavy Ribbon chains down the *a*-axis direction, illustrating the ordering of **L1A** and **L1B** pairs related via the inversion center. These pairs alternate along the *b*-axis direction (solvent molecules were omitted for clarity) **(a)**. The void space generated after in-silico removal of the TCM molecules, along [100] **(b)** and along [010] **(c)**.

Indeed, the CH···O interactions involving **L1A** ligand molecules are established between the methyl and the carbonyl moieties of the ester groups: C21AH21A···O2A (2.446(2) Å/154(2)°) and C23AH23A···O5A (2.644(2) Å/145(1)°) along [001], see Fig.S2a. The **L1B** pairs come together in contact through mutual O···H bonds (Tab.S3), as illustrated in Fig.S3b. These interactions are established between the aliphatic CH moieties of the bispidine core and the terminal ester oxygen atom C6BH6B···O5B (2.367(2) Å/146(1)°), and between the pyridinic aromatic CH involved in a bifurcating interaction with O2B terminal ester- and O3B keto-oxygen atoms C13BH13B···O2B (2.677(2) Å/146(1)°) and C13BH13B···O3B (2.656(2) Å/143(1)°).


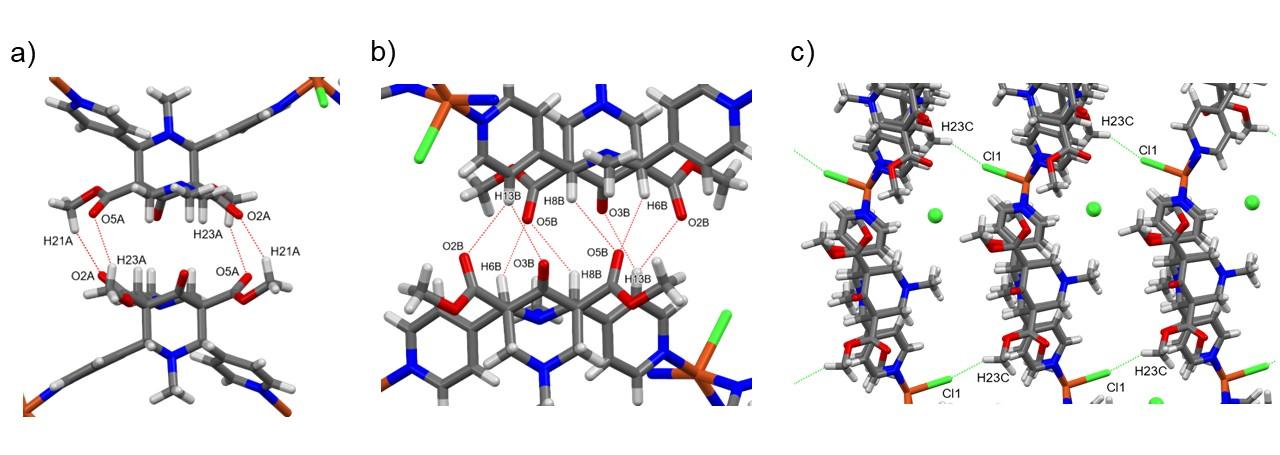


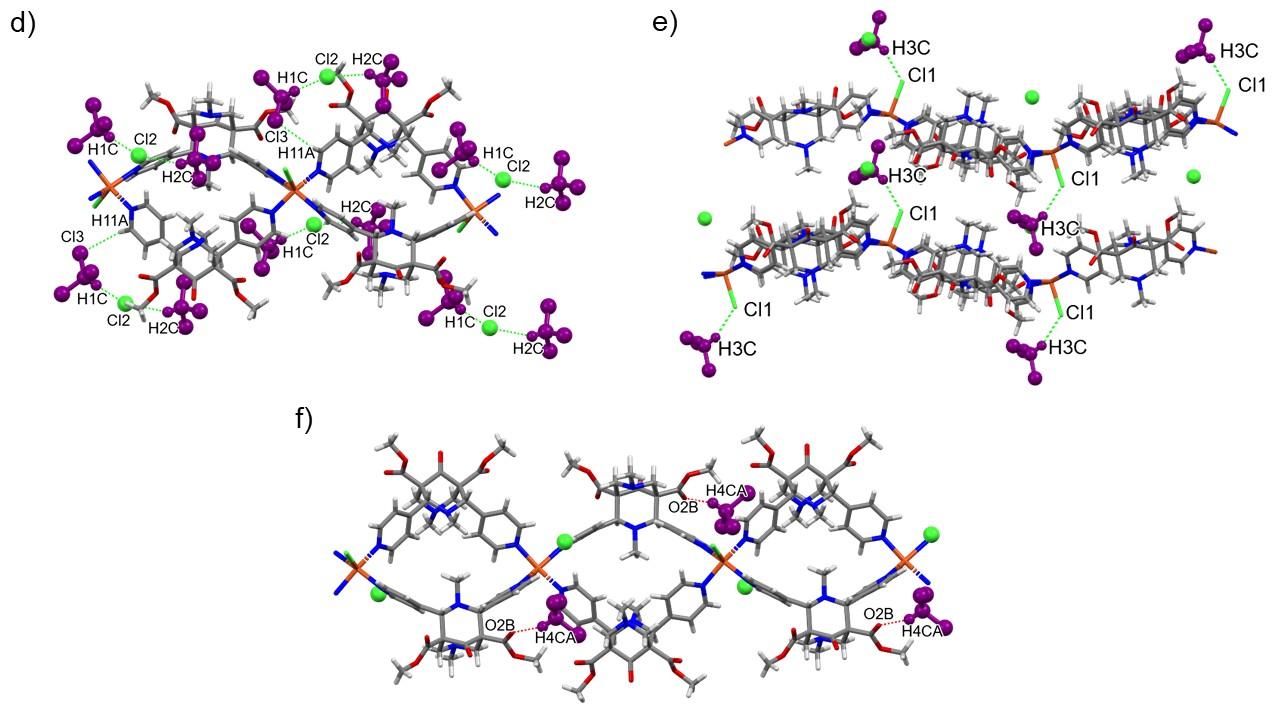


**Figure S2.** Inter-Ribbon chains interactions in **1-TCM^SC^**: views showing CH···O contacts between symmetrically related **L1A** **(a)** and **L1B** **(b)** bispidine ligand molecules; and CH···Cl interactions between the Cu(II)-bound chloride and the ester methyl’s of nearby chains **(c)**. Representation of the solvent-framework interactions: TCM-1 and TCM-2 **(d)**, TCM-3 **(e)** and TCM-4 **(f)**.

Furthermore, the Ribbon-chains interact through H···Cl contact between the ester methyl and the Cu-bound chloride ion C23AH23C···Cl1 (2.808(1) Å/146(1)°), as shown in Fig.S2c.

In contrast, the uncoordinated chloride ion Cl2 engages in five moderate to strong^^[[2]](#footnote-2)^,^[[3]](#footnote-3)^^ Cl···H interactions, see Fig.1c, provided by three aromatic CH (see Tab.S3) along the *c*-axis direction: C12AH12A···Cl2 (2.619(1) Å/167(1)°), C11BH11B···Cl2 (2.524(1) Å/174(1)°) and C5BH5B···Cl2 (2.799(1) Å/155(1)°), as well as by two adjacent TCM molecules oriented alongside the Ribbon units down the *b*-axis: C1CH1C···Cl2 (2.372(1) Å/177(1)°) and C2CH2C···Cl2 (2.665(1) Å/146(1)°), Tab.S3. Notably, only one of the TCM molecules surrounding the uncoordinated Cl2 participates in a unique contact with a nearby chain, involving a chlorine atom and an aromatic CH, C11AH11A···Cl3 (2.859(1) Å/142(1)°). A view of these contacts is provided in Fig.S2d.

The TCM molecule located between the inter-Ribbon forms a CH···Cl contact with the Cu-bound chloride, C3CH3C···Cl1 (2.620(1) Å/152(1)°), see Fig.S2e.

Finally, the fourth TCM molecule, trapped within the V-shaped space created by the terminal ester groups of **L1A** and **L1B** around the metal center (Fig.S2f), interacts with the adjacent Ribbon-chain through a CH···O bond with the ester oxygen atom, C4CAH4CA···O2B (2.304(2) Å/146(1)°).

**Table S3.** Intermolecular interactions’ geometry parameters (Å, °) for **1-TCM^SC^**.

| **Entry** | **D—H···A** | **D—A (Å)** | **H···A (Å)** | **D—H···A (°)** | **Symmetry operation** |
| --- | --- | --- | --- | --- | --- |
| 1 | C21AH21A···O2A | 3.358(4) | 2.446(2) | 154(2) | -x,-y,-z+1 |
| 2 | C23AH23A···O5A | 3.495(4) | 2.644(2) | 145(1) | -x,-y,-z+1 |
| 3 | C6BH6B···O5B | 3.248(3) | 2.367(2) | 146(1) | -x+1,-y+1,-z+1 |
| 4 | C13BH13B···O2B | 3.501(3) | 2.677(2) | 146(1) | -x+1,-y+1,-z+1 |
| 5 | C13BH13B···O3B | 3.461(3) | 2.656(2) | 143(1) | -x+1,-y+1,-z+1 |
| 6 | C23AH23C···Cl1 | 3.663(4) | 2.808(1) | 146(1) | x-1,y,z |
| 7 | C3CH3C···Cl1 | 3.534(3) | 2.620(1) | 152(1) | x,y,z |
| 8 | C12AH12A···Cl2 | 3.553(3) | 2.619(1) | 167(1) | -x+1,y-1/2,-z+3/2 |
| 9 | C11BH11B···Cl2 | 3.470(2) | 2.524(1) | 174(1) | x,y,z |
| 10 | C5BH5B···Cl2 | 3.685(2) | 2.799(1) | 155(1) | -x+1,y+1/2,-z+3/2 |
| 11 | C1CH1C···Cl2 | 3.371(3) | 2.372(1) | 177(1) | x,y,z |
| 12 | C2CH2C···Cl2 | 3.541(4) | 2.665(1) | 146(1) | x,y,z |
| 13 | C11AH11A···Cl3 | 3.655(3) | 2.859(1) | 142(1) | -x,y-1/2,-z+3/2 |
| 14 | C4ACH4CA···O2B | 3.185(5) | 2.304(2) | 146(1) | -x+1,-y+1,-z+1 |
| 15 | C18BH18F···Cl6 | 3.286(3) | 2.811(1) | 111(2) | x,y,z |
| 16 | C23AH23B···Cl11 | 3.746(4) | 2.789(1) | 166(1)° | x-1,+y,+z |
| 17 | C23AH23C···Cl12 | 3.441(5) | 2.814(2) | 122(1) | x-1,+y,+z |

- **1-H_2_O^SC^**

Although **1-TCM^SC^** and **1-H_2_O^SC^** share a similar 1D wavy ribbon topology and comparable geometry of the Cu(II) center and chlorine ions, they display structural differences.

A first distinction of **1-H_2_O^SC^** CP from the original **1-TCM^SC^** is the *anti*-arrangement of ester groups within the bispidine ligands (see Fig.S3b). This variation, along with the influence of the different solvent—H_2_O instead of TCM—results in slightly altered molecular packing.


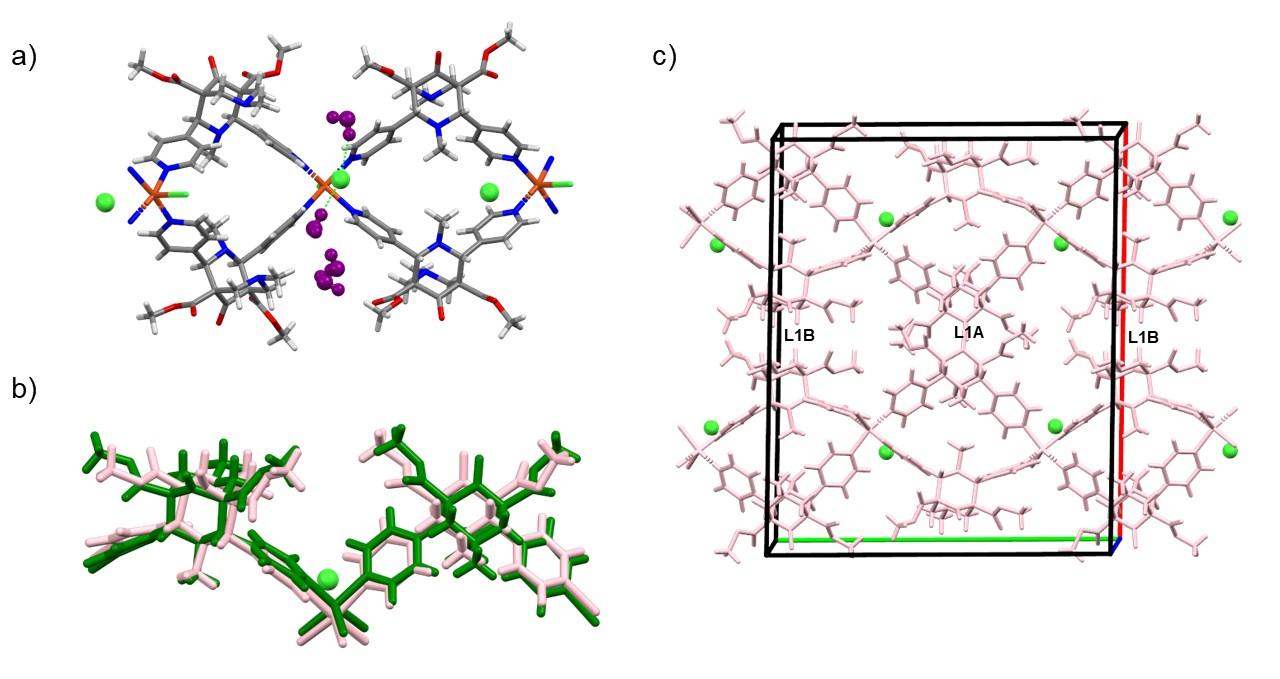


**Figure S3.** View of the molecular structure of **1-H_2_O^SC^** **(a)**, overlay between the asymmetric units of **1-TCM^SC^** (green) and **1-H_2_O^SC^** (pink) depicting the different orientations of the ester groups, which are in *anti* mode in **1-H_2_O^SC^** **(b)**. Projection showing the orientation of the ribbon chains down the *c*-axis direction **(c)**.

Figure S3c depicts the view of the ribbon chains down the [001] direction. These chains interact with one another through mutual O···H contacts.

In the structural model plotted with **L1A** and **L1B**, along the *a*-axis direction, **L1A** from one ribbon-chain and its symmetrically related counterpart **L1A** through *C*_2_ // [010] involves O···H contacts, as shown in Figure S4a, that engage only one of the ester groups, from the terminal methyl group to the C=O oxygen, with the interaction C23AH23B···O2A: 2.714(5)Å/152(1)° (Tab.S4). Furthermore, there is an O···H interaction between the bispidine cores where the aliphatic C-H approaches the keto-oxygen, denoted as C19AH19A···O3A: 2.450(4)Å/164(1)° (Fig.S4a).

In the same manner as **L1A**, symmetrically related **L1B** (via *C*_2_ // [010]) establishes the following interactions, C23BH23E···O2B: 2.581(8)Å/139(1)° in Fig.S4a (in the structural model plotted with **L1D**, the equivalent interaction is C23DH23H···O2D: 2.605(6)Å/151(1)°) and C19BH19D···O3B: 2.629(3)Å/169(1)°, see Tab.S4.

Along the *c*-axis direction, as for the second ester group, **L1A** forms mutual O···H bridging contacts through a bifurcating interaction towards the aliphatic C-H of the bispidine core (shown in Fig.S4b), C6AH6A···O5A: 2.514(6) Å/134(1)° (in the model with **L1C**, C6AH6A···O5C: 2.509(6) Å/132(1)°) and C8AH8A···O5A: 2.317(7) Å/142(1)° (in the model with **L1C**, C8AH8A···O5C: 2.376(7) Å/138(1)°). In addition to a contact between the keto-oxygen O3A and a pyridinic H-atom, C10AH10A···O3A: 2.463(3) Å/156(1)°.

In contrast, the second ester group of **L1B** establishes a unique O···H contact in the *c*-axis direction from O5B towards a pyridinic H-atom, C1BH1B···O5B: 2.662(3) Å/137(1)° (Tab.S4).

The ribbon chains are also bridged via CH···Cl contacts, though this occurs differently in **1-TCM^SC^** and **1-H_2_O^SC^** CPs. For example, in **1-TCM^SC^**, Cl1 engages in unique CH···Cl contact, down to [100], with the parallel chain, and Cl2 interacts only with one chain (see Fig.S2c). Conversely, in **1-H_2_O^SC^**, Cl1 does not participate in inter-chain interactions, while Cl2 holds two chains along [001], forming three interactions with one chain via C1AH1A···Cl2: 2.898(1) Å/134(1)°, C12AH12A···Cl2: 2.631(1) Å/163(1)° and C5BH5B···Cl2: 2.722(1) Å/134(1)°, and bridging the parallel chain via a unique interaction, C4BH4B···Cl2: 2.835(1) Å/149(1)°.

As in **1-TCM^SC^**, the unbound Cl2 ion forms robust ion-dipole interactions with two co-crystallized water molecules in **1-H_2_O^SC^**, labeled as “W2” and “W3”, O2WH2WB···Cl2 (2.262(61) Å/169(5)°) and O3WH2WA···Cl2 (2.518(84) Å/159(6)°), see Figure S4d.

While W3 interacts exclusively with Cl2, W2 is involved in several O···H interactions between the ribbon chains running parallelly to the *c*-axis. The oxygen atom of W2, acting as an acceptor, engages in three interactions: two with H-atoms from one ribbon chain via a terminal methyl group of the bispidine core, C18BH18F···O2W (2.556(5) Å/141(1)°), and via an aromatic C-H, C11BH11B···O2W (2.549(3) Å/124(1)°); in addition to an interaction with another water molecule (W1), O1WH1WB···O2W (1.977(67) Å/163(6)°), Fig.S4d.

Alongside the parallel ribbon chain, W2 (acting as a donor) forms another O···H interaction with the keto-oxygen atom O3B, O2WH2WA···O3B (2.164(43) Å/166(3)°).


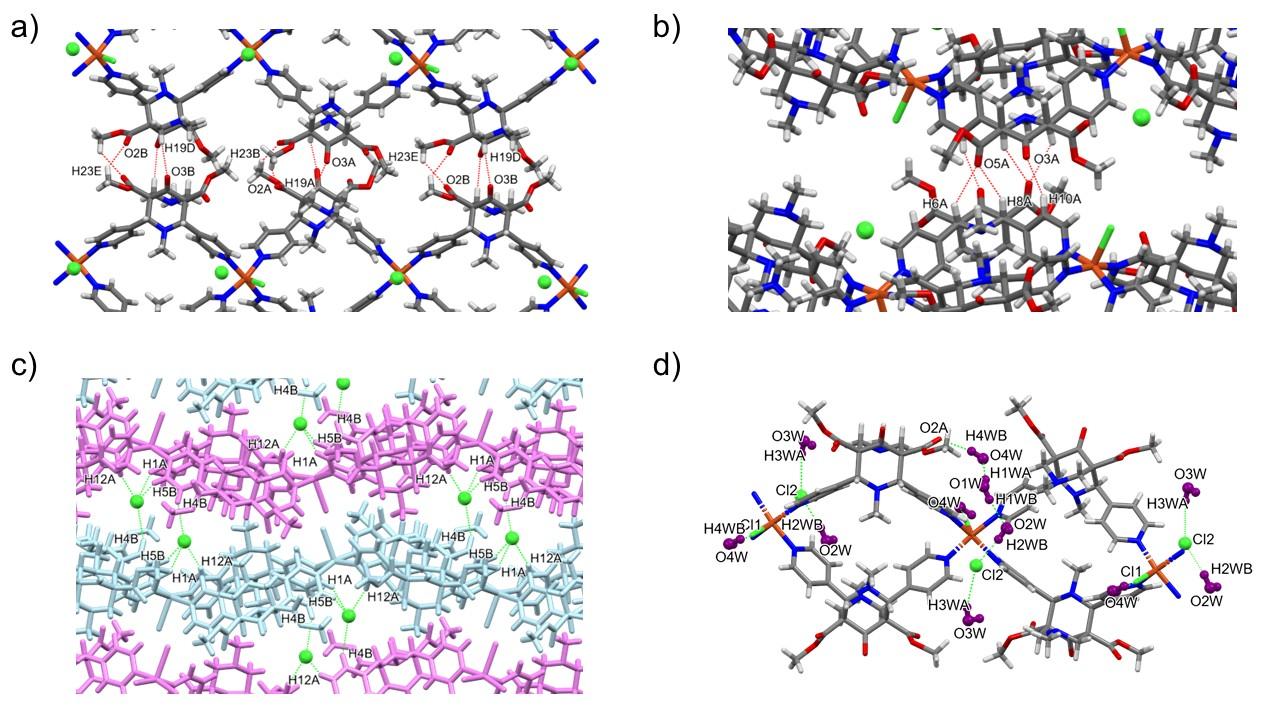


**Figure S4.** Views of the intermolecular interactions in the crystal structure of **1-H_2_O^SC^**. Mutual O···H interactions between symmetrically related **L1A**, and **L1B** bispidine ligands along the [010] direction **(a)**. O···H contacts between **L1A** pairs along the [001] direction **(b)**. Projection showing the CH···Cl2 interactions between the ribbon chains down to the [001] direction. The main intermolecular interactions between water molecules and the ribbon chain **(d)**.

**Table S4.** Intermolecular interactions’ geometry parameters (Å, °) for **1-H_2_O^SC^**, **C** and **D** indices refer to the second disordered sites in the ester groups.

| **Entry** | **D—H···A** | **D—A (Å)** | **H···A (Å)** | **D—H···A (°)** | **Symmetry operation** |
| --- | --- | --- | --- | --- | --- |
| 1 | C23AH23B···O2A | 3.607(9) | 2.714(5) | 152(1) | -x+1,y,-z+1/2 |
| 2 | C19AH19A···O3A | 3.411(7) | 2.450(4) | 164(1) | -x+1,y,-z+1/2 |
| 3 | C23BH23E···O2B | 3.383(14) | 2.581(8) | 139(1) | -x+1,y,-z+1/2 |
| 4 | C23DH23H···O2D | 3.497(14) | 2.605(6) | 151(1) | -x+1,y,-z+1/2 |
| 5 | C19BH19D···O3B | 3.606(5) | 2.629(3) | 169(1) | -x+1,y,-z+1/2 |
| 6 | C6AH6A···O5A | 3.290(6) | 2.514(6) | 134(1) | -x+1,-y+1,-z |
| 7 | C6AH6A···O5C | 3.265(6) | 2.509(6) | 132(1) | -x+1,-y+1,-z |
| 8 | C8AH8A···O5A | 3.167(7) | 2.317(7) | 142(1) | -x+1,-y+1,-z |
| 9 | C8AH8A···O5C | 3.187(7) | 2.376(7) | 138(1) | -x+1,-y+1,-z |
| 10 | C10AH10A···O3A | 3.355(4) | 2.463(3) | 156(1) | -x+1,-y+1,-z |
| 11 | C1BH1B···O5B | 3.420(5) | 2.662(3) | 137(1) | x,-y,z-1/2 |
| 12 | C1AH1A···Cl2 | 3.620(5) | 2.898(1) | 134(1) | x,y,z |
| 13 | C12AH12A···Cl2 | 3.549(4) | 2.631(1) | 163(1) | -x+3/2,y+1/2,-z+1/2 |
| 14 | C5BH5B···Cl2 | 3.454(4) | 2.722(1) | 134(1) | -x+3/2,y-1/2,-z+1/2 |
| 15 | C4BH4B···Cl2 | 3.682(5) | 2.835(1) | 149(1) | x,-y,1/2+z |
| 16 | O2WH2WB···Cl2 | 3.095(5) | 2.262(61) | 169(5) | x,y,z |
| 17 | O3WH3WA···Cl2 | 3.320(7) | 2.518(84) | 159(6) | x,y,z |
| 18 | C18BH18F···O2W | 3.377(8) | 2.556(5) | 141(1) | x,y,z |
| 19 | C11BH11B···O2W | 3.184(5) | 2.549(3) | 124(1) | x,y,z |
| 20 | O1WH1WB···O2W | 2.792(7) | 1.977(67) | 163(6) | x,y,z |
| 21 | O2WH2WA···O3B | 2.988(5) | 2.164(43) | 166(3) | x,-y,z-1/2 |
| 22 | C23AH23C···O1W | 3.427(7) | 2.483(4) | 162(1) | x,y,z |
| 23 | O1WH1WA···O4W | 2.808(6) | 1.993(29) | 163(1) | x,y,z |
| 24 | O4WH4WB···O2A | 2.950(5) | 2.116(42) | 171(4) | x,y,z |
| 25 | O4WH4WA···Cl1 | 3.226(4) | 2.418(34) | 162(3) | -x+1/2+1,-y+1/2,-z+1 |

W1 establishes a total of three OH interactions. One of these interactions is with W2, as mentioned earlier. Additionally, two other interactions occur: one with the terminal methyl of the ester group of **L1A** from one side, C23AH23C···O1W (2.483(4) Å/162(1)°), and another one with water molecule “W4” on the other side, O1WH1WA···O4W (1.993(29) Å/163(1)°), Fig.S4d. The latter also interacts with the framework through strong interactions. An O···H contact occurring with the terminal oxygen of the ester group of **L1A**, O4WH4WB···O2A (2.116(42) Å/171(4)°) (in Fig.S4d); and with another ribbon chain through a Cl···H one with the bound Cl1 ion, O4WH4WA···Cl1 (2.418(34) Å/162(3)°).


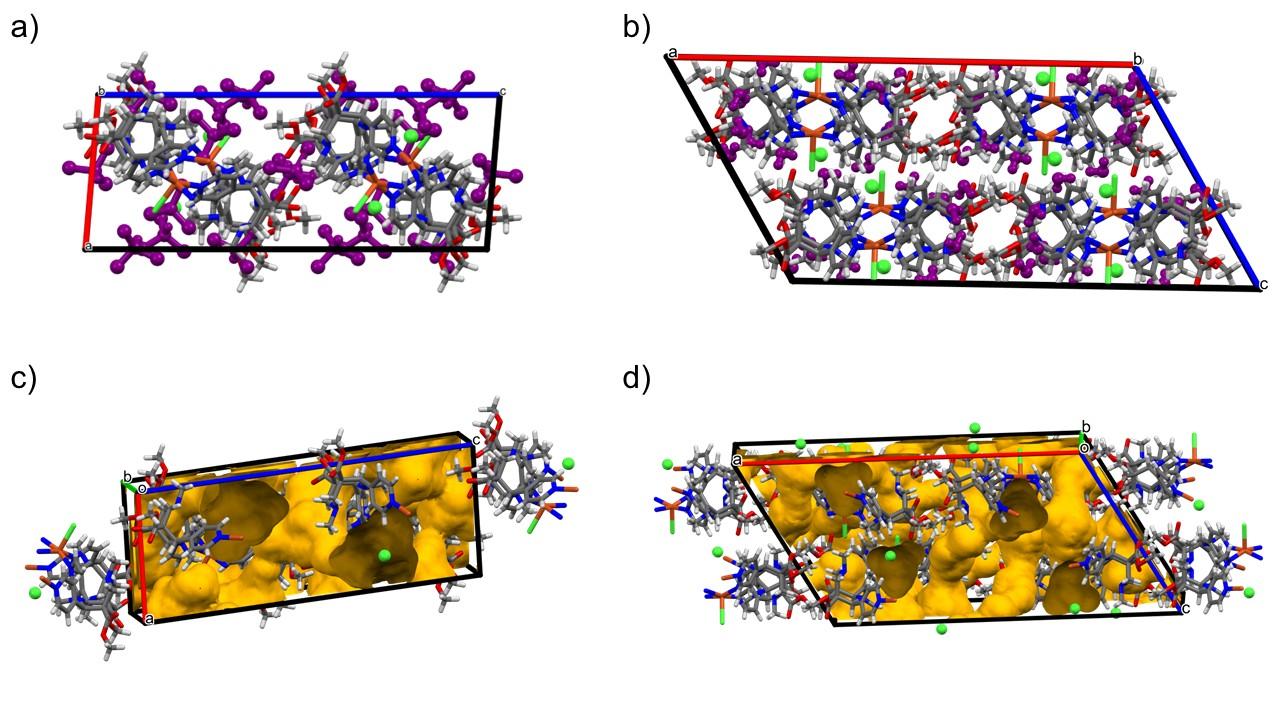


**Figure S5.** Crystallographic projections along the *b*-axis of the unit cells of **1-TCM^SC^** **(a)** and **1-H_2_O^SC^** **(b)** illustrate the varying solvent distributions within the ribbon chains. A view showing the void’s shape in **1-TCM^SC^** **(c)** and **1-H_2_O^SC^** **(d)**.

- **1-MeCN^SC^**

**1-MeCN^SC^** Mutual CH···O interactions via the bispidine ligand (listed in Table S5) along the *a*-axis direction, as shown in Figure S6a, contribute to build up the crystal packing. Specifically, the terminal ester oxygens are involved in one bifurcating CH···O interactions with two aliphatic CH of the bispidine core placed in the successive layer, C6AH6A···O2A (2.487(1) Å/138(1)°) and C8AH8A···O2A (2.358(1) Å/142(1)°). Additionally, at the extremities, the keto oxygen atom interacts with the aromatic hydrogen one, C4AH4A···O3A (2.522(1) Å/146(2)°).

The acetonitrile molecules are located within the inter-sheets spaces crystal lattice and act as H-bond acceptor being involved in CH···N interaction (Tab.S5) which occurs between the nitrile functional group and the bispidine aromatic CH (C10AH10A···N1AC, 2.619(2) Å/140(1)°), as depicted in Figure S5b. Their in-silico removal evidences that they occupy large, isolated voids that account for 13.7 % of the total unit cell volume, as illustrated in Fig.S6c.


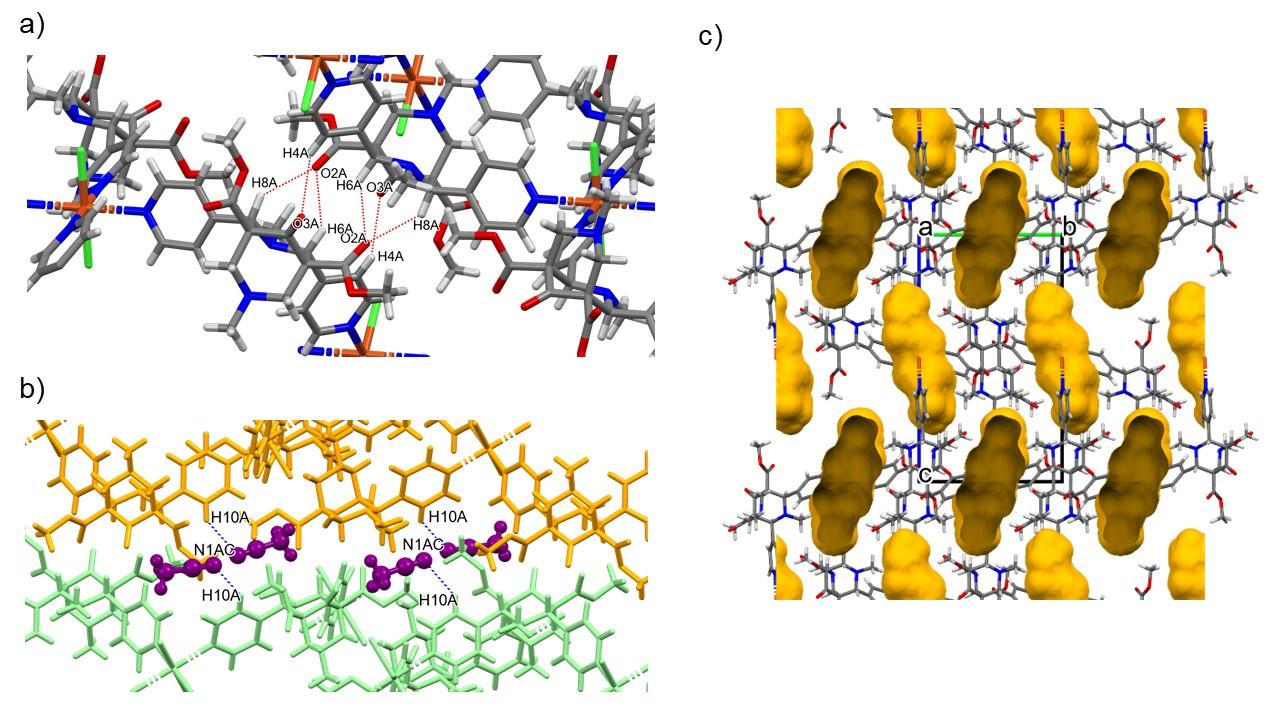


**Figure S6.** The O···H inter-sheets interactions in **1-MeCN^SC^** **(a)**. Weak CH···N interactions between the MeCN molecules (depicted in Ball&Stick, purple) and the adjacent sheets **(b)**. Packing views along [100] showing the voids spaces, generated after *in*-*silico* removal of MeCN molecules **(c)**.

**Table S5.** Intermolecular interactions’ geometry parameters (Å, °) for **1-MeCN^SC^**.

| **Entry** | **D—H···A** | **D—A (Å)** | **H···A (Å)** | **D—H···A (°)** | **Symmetry operation** |
| --- | --- | --- | --- | --- | --- |
| 1 | C6AH6A···O2A | 3.307(2) | 2.487(1) | 138(1) | -x+1,-y+1,-z+1 |
| 2 | C8AH8A···O2A | 3.211(2) | 2.358(1) | 142(1) | -x+1,-y+1,-z+1 |
| 3 | C4AH4A···O3A | 3.333(2) | 2.522(1) | 146(2) | -x+1,-y+1,-z+1 |
| 4 | C10AH10A···N1AC | 3.405(2) | 2.619(2) | 140(1) | -x+1/2,y-1/2,-z+1/2 |

- - - 1. **Hirshfeld surface analysis**


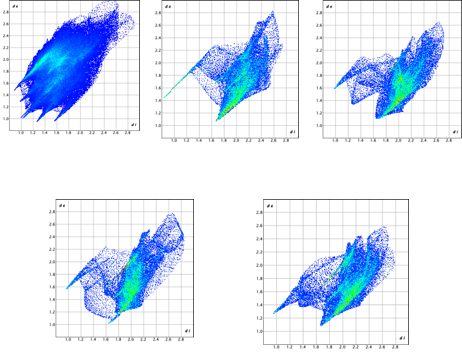


**Figure S7.** Fingerprint plots of the CP and TCM molecules in **1-TCM^SC^**. Top left: the CP; top middle: the TCM molecule more tightly bound to the chloride ion Cl1; top right: the TCM molecule less tightly bound to the chloride ion Cl1; bottom left : the TCM molecule interacting with the Cu-bound chloride Cl2; bottom right: the TCM molecule H-bonded to an O=C provided by the CP.


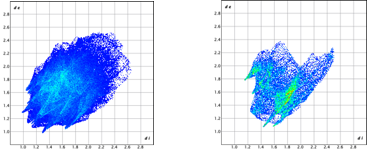


**Figure S8.** Fingerprint plots of the CP and MeCN molecules in **1-MeCN^SC^**. Top left: the CP; top right: the MeCN.

- - - 1. **Variable temperature single crystal X-ray diffraction (VT SC-XRD)**

The thermal expansion coefficients (TECs) for **1-TCM^SC^** and **1-MeCN^SC^** CPs calculated based on the change in linear parameters and volume, α and β, respectively, using the cell parameter values at 100 K as the reference, are given below.

**Table S6.** α and β thermal expansion coefficients (TECs) of **1-TCM^SC^**.

| *T* (K) | α_a_ | α_b_ | α_c_ | β |
| --- | --- | --- | --- | --- |
| 150 | 1.1287E-04 | -6.5895E-05 | -5.2963E-05 | 2.4067E-03 |
| 200 | 1.2976E-04 | -3.8583E-05 | -2.5736E-05 | 3.9827E-03 |
| 250 | 1.5468E-04 | -1.6763E-05 | -6.2164E-06 | 4.6875E-03 |
| 270 | 1.6088E-04 | -1.3771E-05 | -3.7298E-06 | 4.7262E-03 |
| 290 | 1.7543E-04 | -2.9662E-06 | 4.3187E-06 | 4.6158E-03 |
| 310 | 1.8675E-04 | 1.8579E-06 | 1.4031E-05 | 4.3224E-03 |
| 330 | 1.7177E-04 | -5.6546E-06 | -1.6217E-07 | 3.9101E-03 |
| 350 | 1.6536E-04 | 6.9363E-06 | 2.9838E-07 | 3.3678E-03 |
| 370 | 2.1028E-04 | 3.5003E-05 | -4.2962E-05 | 3.4293E-03 |
| 390 | 2.1208E-04 | 4.3800E-05 | -1.0418E-05 | 2.9884E-03 |
| 400 | 1.9086E-04 | 5.2456E-05 | 3.0957E-05 | 2.1910E-03 |

**Table S7.** α and β thermal expansion coefficients (TECs) of **1-MeCN^SC^**.

| *T* (K) | α_a_ | α_b_ | α_c_ | β |
| --- | --- | --- | --- | --- |
| 150 | 4.7673E-05 | 1.1691E-04 | 3.4610E-05 | 1.1377E-04 |
| 200 | 4.4951E-05 | 7.4695E-05 | 3.6024E-05 | 1.1377E-04 |
| 250 | 5.0738E-05 | 6.3357E-05 | 3.6137E-05 | 1.2088E-04 |
| 300 | 4.9925E-05 | 5.7218E-05 | 3.2563E-05 | 1.1831E-04 |
| 310 | 5.1480E-05 | 5.8929E-05 | 3.8951E-05 | 1.2980E-04 |
| 320 | 5.0633E-05 | 5.6600E-05 | 3.9485E-05 | 1.2821E-04 |
| 330 | 5.1533E-05 | 5.3953E-05 | 3.5543E-05 | 1.2246E-04 |
| 340 | 5.3022E-05 | 5.4839E-05 | 3.9464E-05 | 1.2954E-04 |
| 350 | 5.3791E-05 | 5.4047E-05 | 4.1844E-05 | 1.3257E-04 |
| 360 | 5.3238E-05 | 5.4269E-05 | 3.8566E-05 | 1.2930E-04 |
| 370 | 5.4986E-05 | 5.1246E-05 | 4.0568E-05 | 1.3051E-04 |
| 380 | 5.6139E-05 | 5.3048E-05 | 4.4516E-05 | 1.3826E-04 |
| 390 | 5.6371E-05 | 4.8862E-05 | 4.4494E-05 | 1.3390E-04 |
| 400 | 6.9444E-05 | 5.2446E-05 | 4.5935E-05 | 1.5486E-04 |

- - - 1. **Hot-stage microscopy**


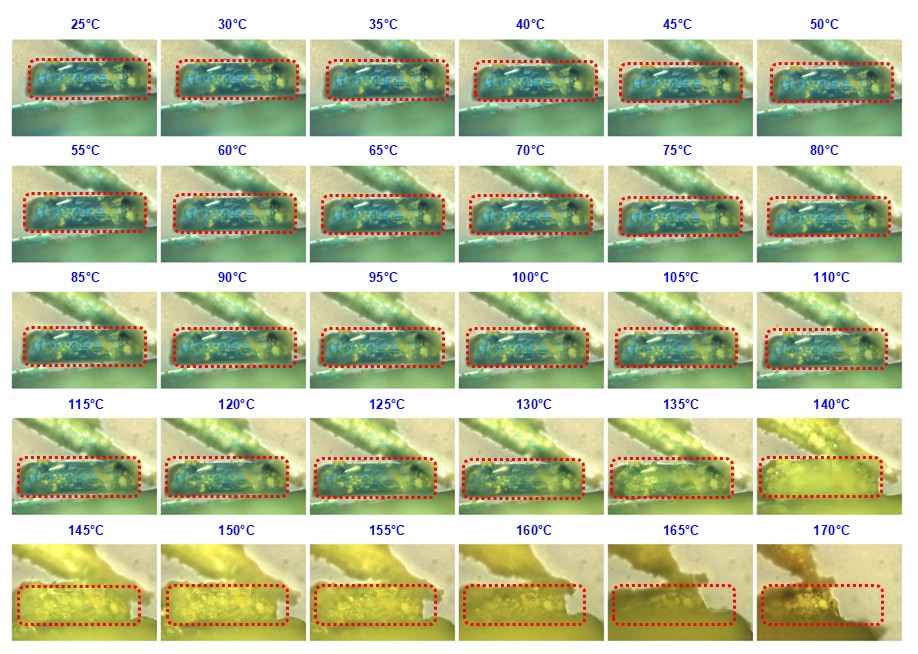


**Figure S9.** Hot-stage micro-images in transmission mode of **1-TCM^SC^** CP heated in the temperature range from 25 to 170°C (298-443 K). Starting at 135°C, the crystal sample gradually turned yellow and began melting around 160°C.


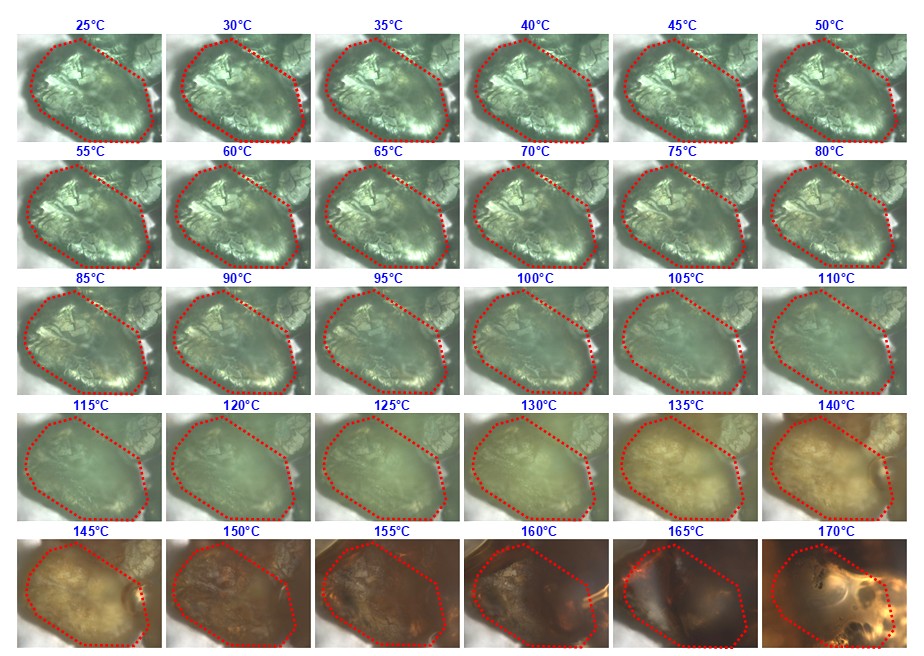


**Figure S10.** Hot-stage microimages in transmission mode of **1-MeCN^SC^** CP heated in the temperature range from 25 to 170°C (298-443 K). A change in color becomes evident at 130°C, and melting of the sample begins approximately from 140°C.

- - - 1. **Powder X-ray diffraction (P-XRD) and thermogravimetry (TG) coupled with FTIR spectroscopy of evolved gase**s


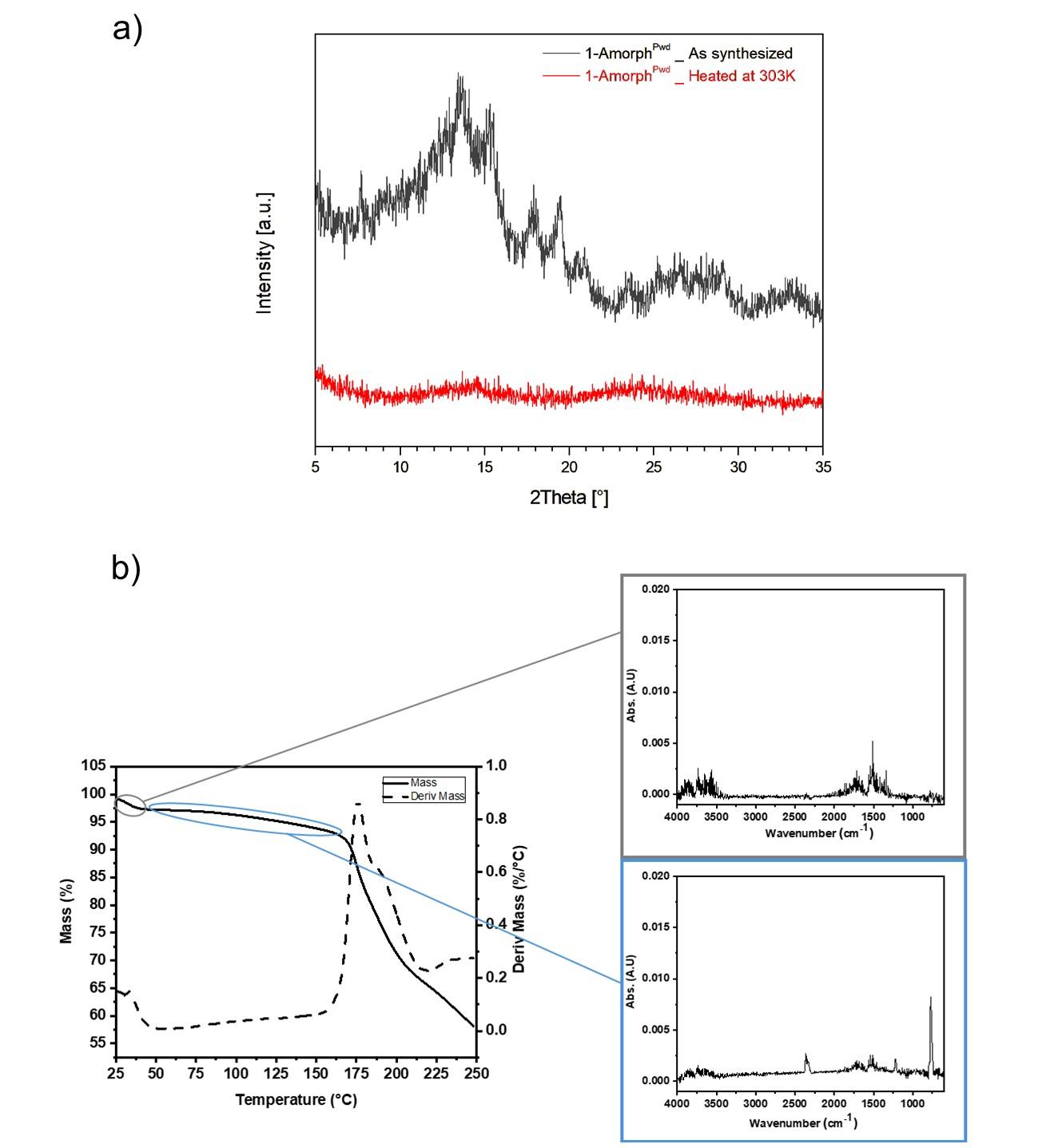


**Figure S11.** Experimental P-XRD patterns of **1-Amorph^Pwd^** as synthesized (black) and heated at 303 K **(a)**. Mass loss profile (TG curve, solid line) and its derivative (dmass%/dT, DTG, dashed line) of **1-Amorph^Pwd^** obtained by TGA-FTIR experiments **(b)**. The FTIR spectra of evolved gases (shown in the inserts) highlight the presence of water below 50 °C (corresponding to a mass loss of about 3%) and the presence of TCM in the range 75-150°C, corresponding to another 3% of mass loss.


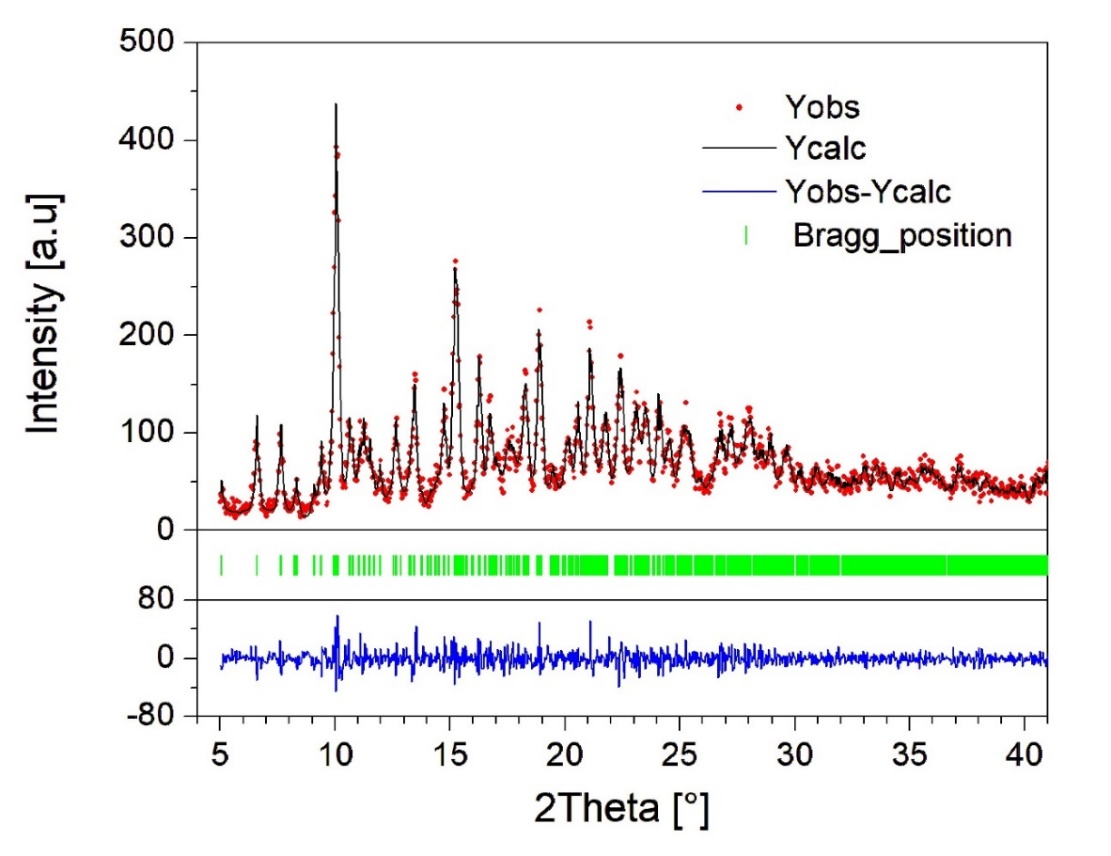


**Figure S12.** Pawley refinement results for **1-TCM^Pwd^**. Red circles are experimental data (Yobs) of **1-Amorph^Pwd^** exposed to TCM vapors for 02 weeks, black line is the calculated pattern (Ycalc) from SC-XRD data of **1-TCM^SC^** at room temperature, green vertical lines are the Bragg positions, and the blue line is the difference between experimental and calculated intensities (Yobs-Ycalc).

Figure S12 shows the refined XRD profile of **1-TCM^Pwd^**, generated using the FullProf Suite program.^[[4]](#footnote-4)^ The refinement of the XRD profile utilized the Pawley method,^[[5]](#footnote-5)^ applying a constant scale factor along with the unit cell parameters, peak shape (pseudo-Voigt approach), zero-error, and background parameters. The results reveal that **1-Amorph^Pwd^** nearly crystallized into a single phase of the **1-TCM^Pwd^** monoclinic-type structure (*P*2_1_/*c*) after TCM adsorption, without any impurity phases. The intensity differences between the observed and calculated peaks are attributed to the preferential orientation. The refined lattice parameters *a*, *b*, *c* and *β* are found to be 10.775 Å, 23.085 Å, 26.874 Å, and 95.490°, respectively, close to the values inferred from SC-XRD refinement (room temperature data: *a* = 10.788 Å, *b* = 23.151 Å, *c* = 26.881 Å and *β* = 95.613°). Overall, the reliability factors, which include agreement factors of profile *R*_p_ (12.0), weighted profile factor *R*_wp_ (15.5), structure factor *R*_F_ (0.713), and goodness of fit *χ*^2^ (1.110), show reasonable agreement between the observed and calculated P-XRD data.

**
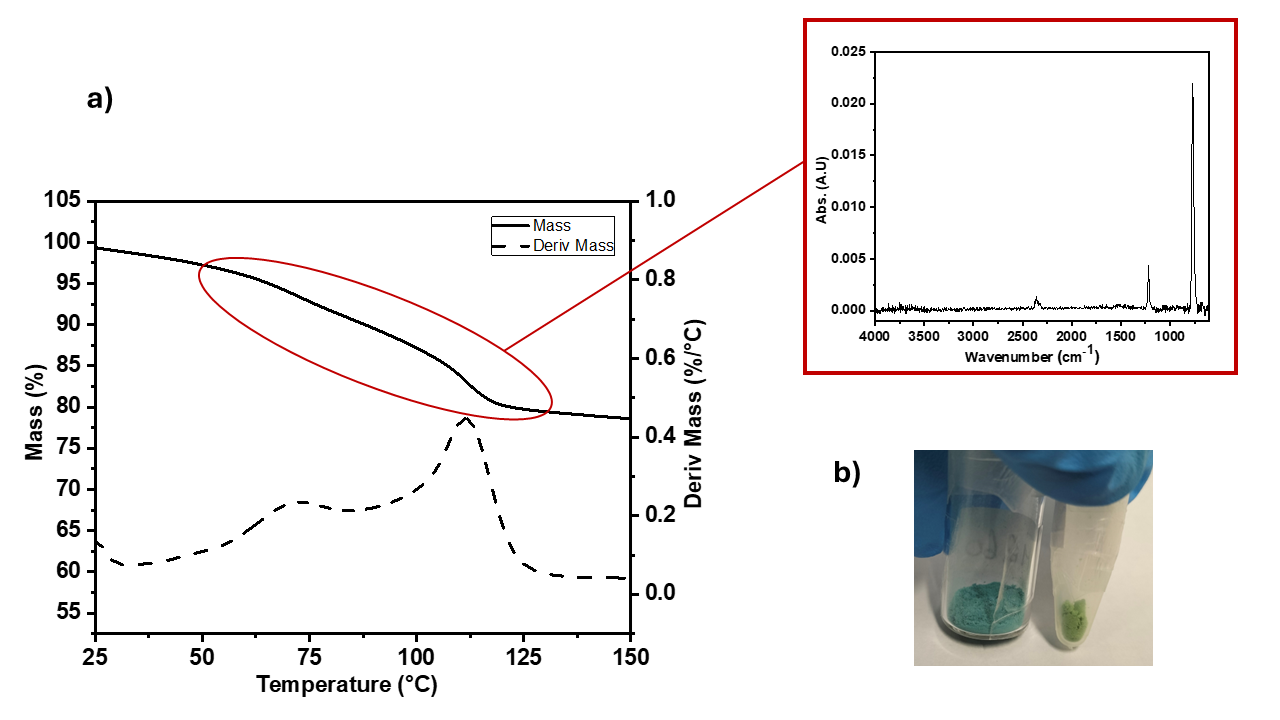
**

**Figure S13**. TG curve (solid line) and DTG curve (dashed line) of **1-TCM^Pwd^** obtained by High Res mode TGA-FTIR experiments **(a)**. The FTIR spectra of evolved gases (shown in the insert) highlight the presence of TCM in the temperature range 25-150 °C, corresponding to a total mass loss of about 22%. The thermal profile shows two desorption steps, which may be attributed to the loss of 3 TCM molecules per formula unit of the CP. Photo of **1-TCM^Pwd^** before and after the thermal scan **(b)**.


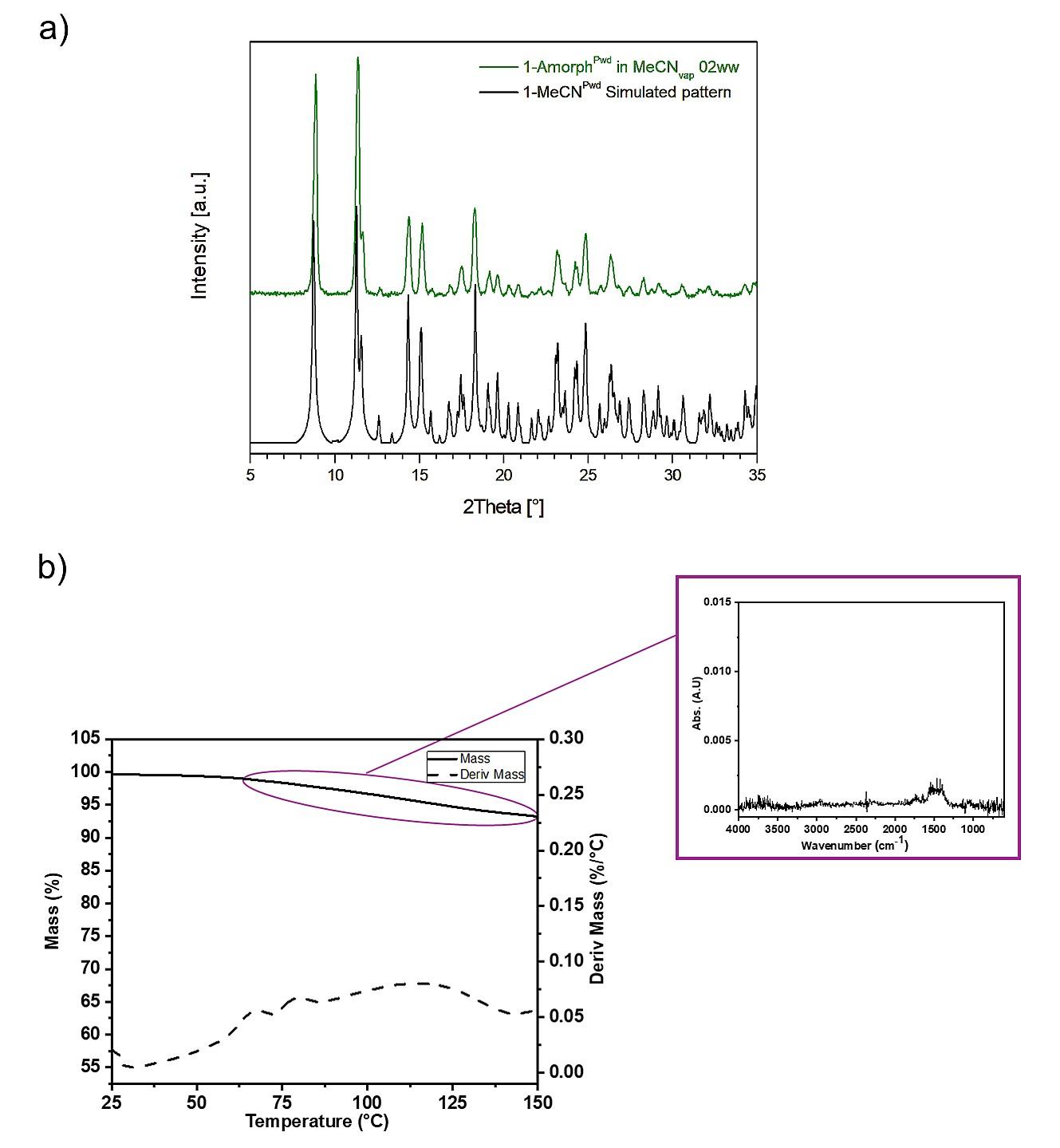


**Figure S14.** Comparison between the experimental (green) and simulated (black) P-XRD patterns of **1-MeCN^Pwd^** **(a)**. TG curve (solid line) and DTG curve (dashed line) of **1-MeCN^Pwd^** obtained by High-Res mode TGA-FTIR experiments **(b)**. The FTIR spectra of evolved gases (shown in the insert) suggest the presence of MeCN in the temperature range 50-150 °C corresponding to a total mass loss of 6%. Elemental analysis of the sample of expected composition [Cu(**L1**)_2_Cl_2_]. Anal. Calcd. for C_46_H_52_Cl_2_CuN_8_O_10_: C, 54.63; H, 5.18; N, 11.08; found C, 55.03; H, 5.34; N, 11.39.


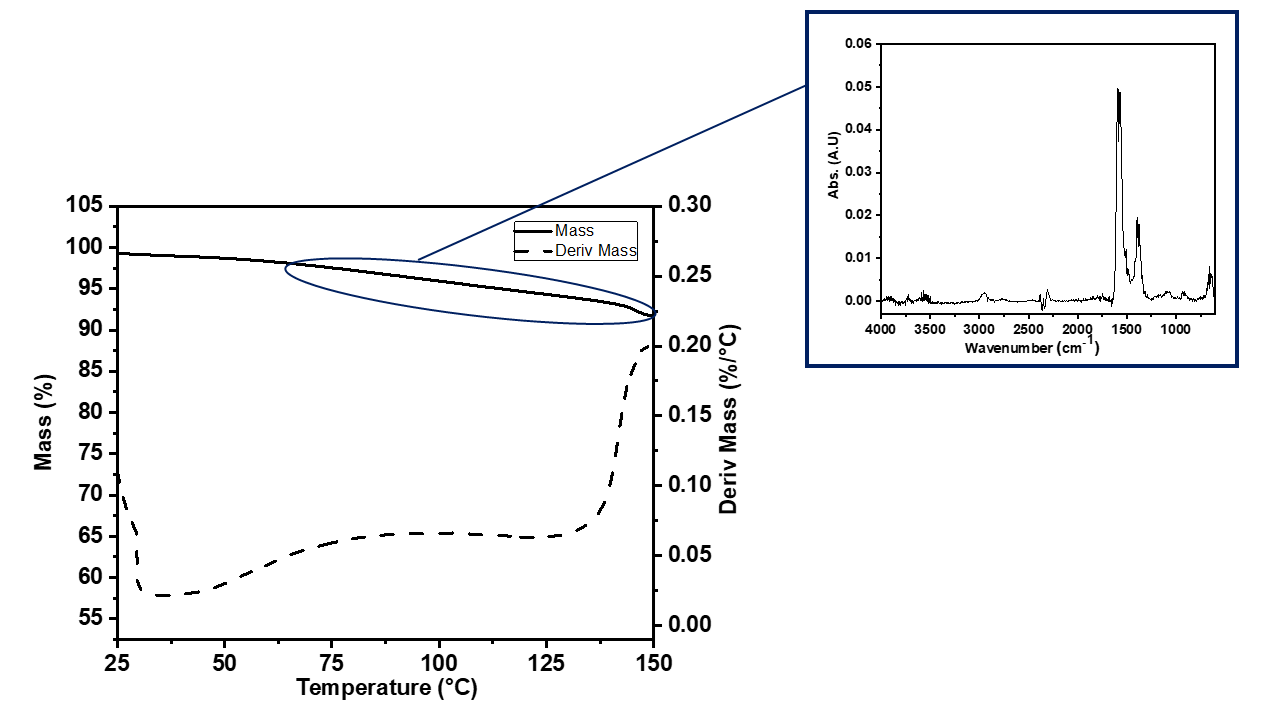


**Figure S15.** TG curve (solid line) and DTG curve (dashed line) of **1-Amorph^Pwd^** exposed to MeNO_2_ vapors obtained by High Res mode TGA-FTIR experiments **(a)**, obtained by TGA-FTIR. The FTIR spectra of evolved gases (shown in the insert) highlight the presence of MeNO_2_ above 50°C, corresponding to a total mass loss of 8%. The DTG curve shows a sharp increase at about 140°C, corresponding to a mass loss step that is probably incomplete by 150°C. This could lead to an underestimation of the overall amount of the adsorbed MeNO_2_, but could also be related to a simultaneous early degradation of the polymer caused by the interaction with MeNO_2_ that would decrease its thermal stability.


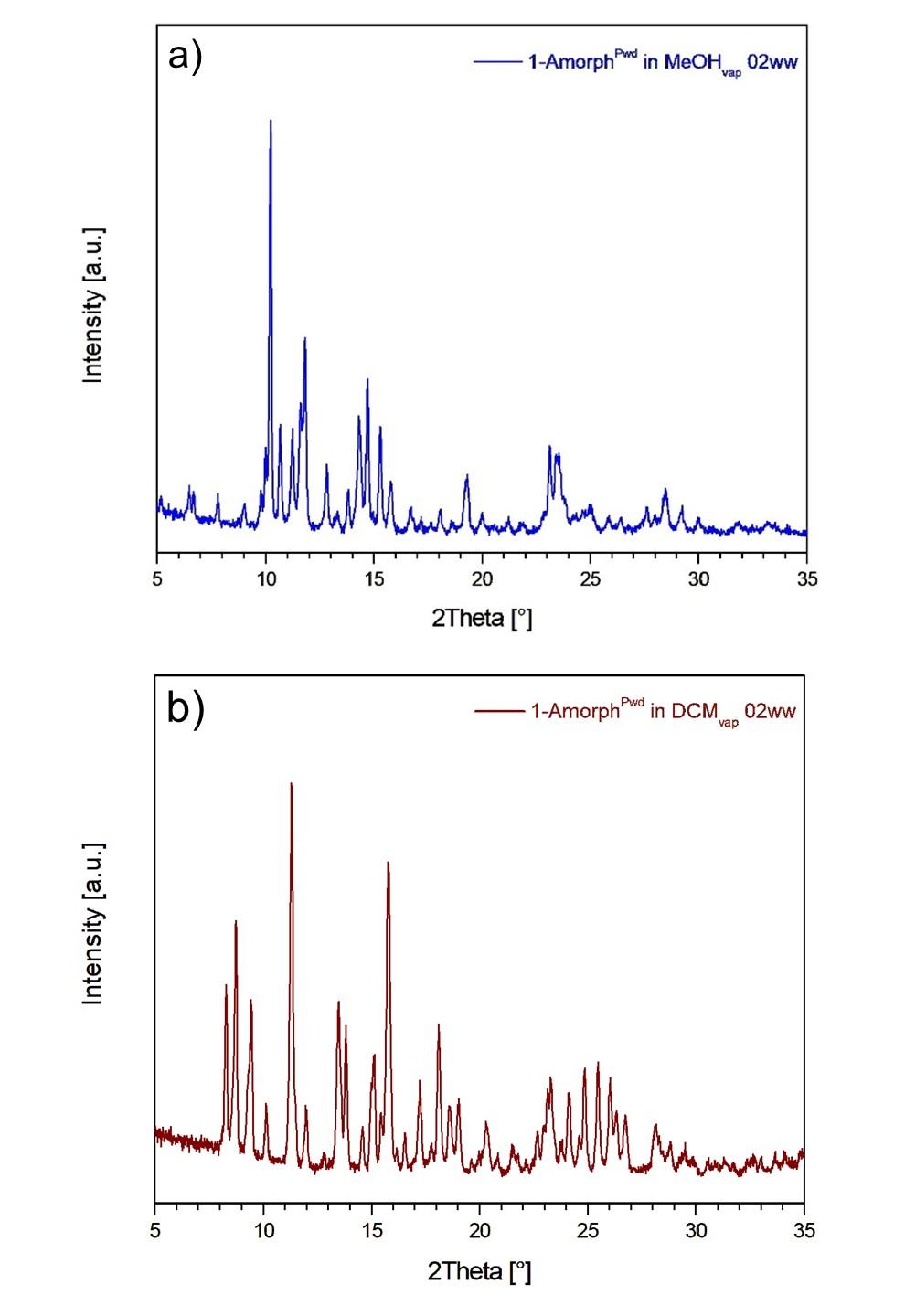


**Figure S16.** Experimental P-XRD data of **1-Amorph^Pwd^** powder samples exposed to DCM **(a)** and MeOH **(b)** vapors for two weeks, demonstrating a transition from an amorphous to crystalline states.


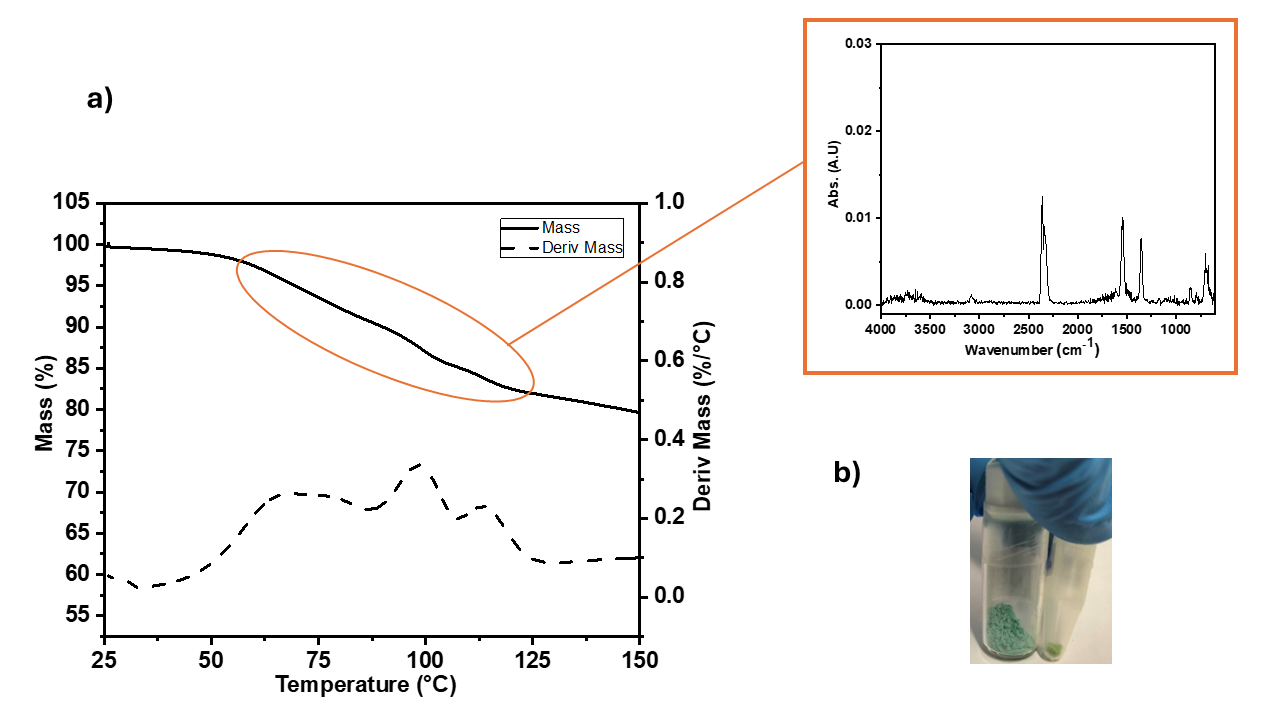


**Figure S17.** TG curve (solid line) and DTG curve (dashed line) of **1-Amorph^Pwd^** exposed to NB vapors obtained by High Res mode TGA-FTIR experiments **(a)**. The FTIR spectra of evolved gases (shown in the insert) highlight the presence of NB in the temperature range 25-150 °C, corresponding to a total mass loss of 22%. The thermal profile shows three desorption steps. Photo of **1-Amorph^Pwd^** exposed to NB vapors before and after the thermal scan **(b)**.


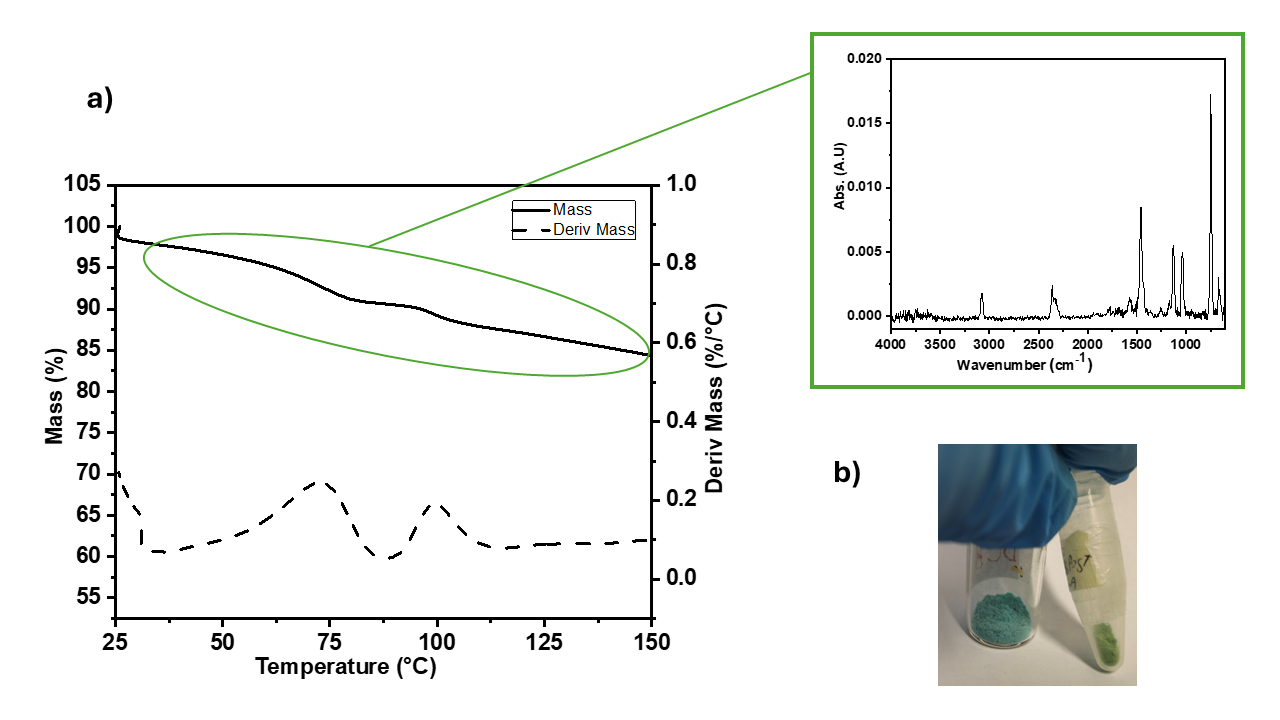


**Figure S18.** TG curve (solid line) and DTG curve (dashed line) of **1-Amorph^Pwd^** exposed to 1,2-DCB vapors obtained by High Res mode TGA-FTIR experiments **(a)**. The FTIR spectra of evolved gases (shown in the insert) highlight the presence of 1,2-DCB in the temperature range 25-150 °C, corresponding to a total mass loss of 15%. The thermal profile shows two desorption processes, which may be attributed to the loss of 2 1,2-DCB molecules per formula unit. Photo of **1-Amorph^Pwd^** exposed to 1,2-DCB vapors before and after the thermal scan **(b)**.


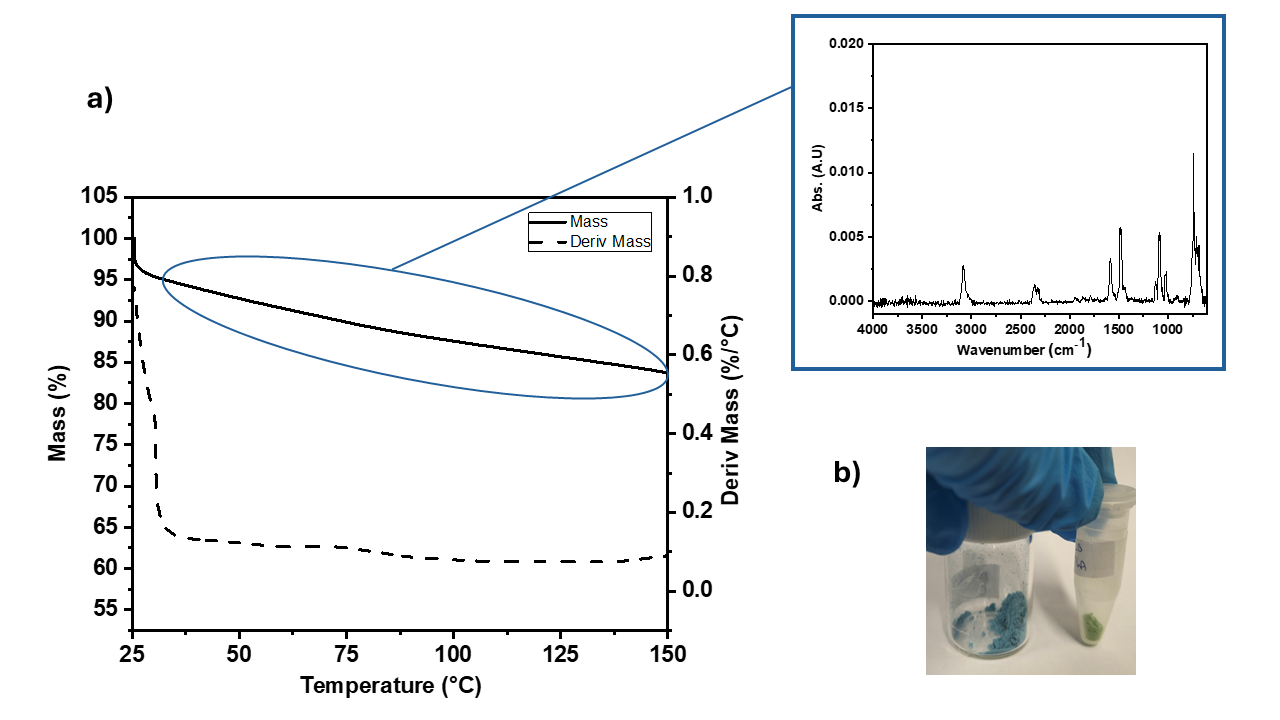


**Figure S19.** TG curve (solid line) and DTG curve (dashed line) of **1-Amorph^Pwd^** exposed to ClBz vapors obtained by High Res mode TGA-FTIR experiments **(a)**. The FTIR spectra of evolved gases (shown in the insert) highlight the presence of ClBz in the temperature range 25-150 °C, corresponding to a total mass loss of 15%. A continuous mass loss which may be attributed to the loss of 2 ClBz molecules, per formula unit is observed. Photo of **1-Amorph^Pwd^** exposed to ClBz vapors before and after the thermal scan **(b)**.


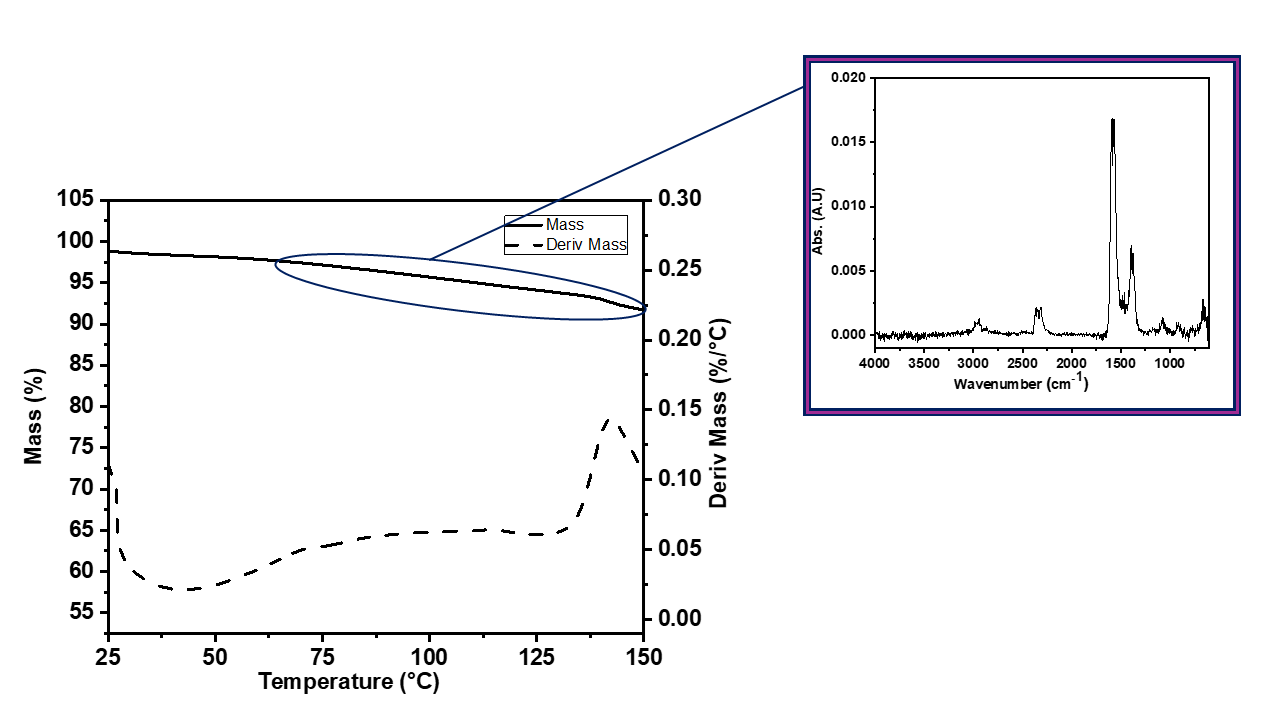


**Figure S20.** TG curve (solid line) and DTG curve (dashed line) of **1-Amorph^Pwd^** exposed to MeCN/MeNO_2_ vapors obtained by High Res mode TGA-FTIR experiments. The FTIR spectra of evolved gases (shown in the insert) show the clear presence of MeNO_2_ above 50°C, corresponding to a total mass loss of 8%, while the presence of MeCN was not detected (absent or below the instrument’s detectability limit).

- - - 1. **Variable temperature powder X-ray diffraction (VT P-XRD)**


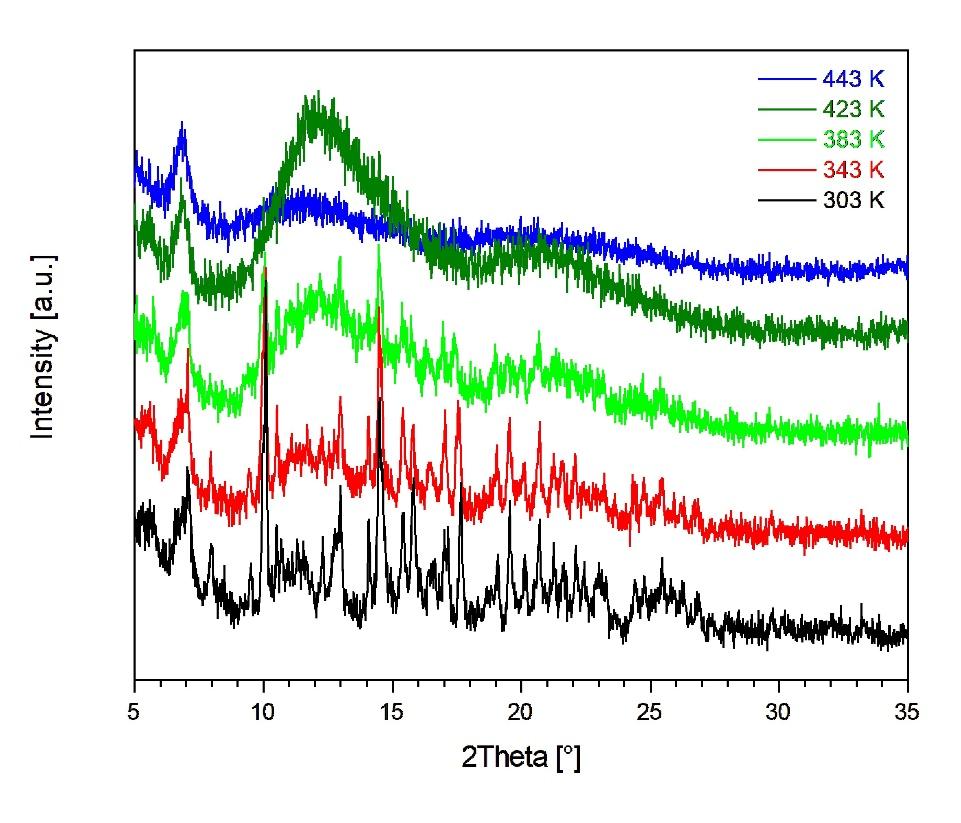


**Figure S21.** VT P-XTD patterns of **1-TCM^Pwd^** show phase stability up to 383 K. At 423 K, the sample becomes amorphous, and at 443 K, it starts to melt. The peak observed between 2*θ* from 5.9 to 7.3° in all the patterns is attributed to the sample holder.


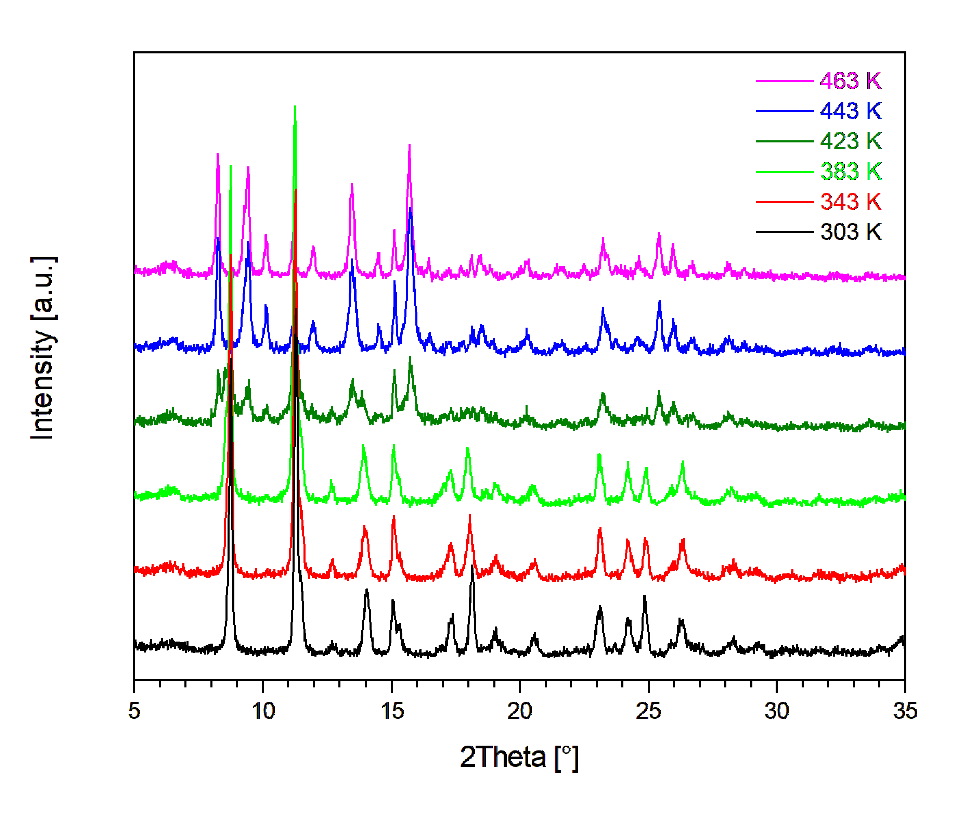


**Figure S22.** VT P-XRD patterns of **1-MeCN^Pwd^**, indicating phase stability up to 383 K. Between 423 and 463 K, the sample transitioned progressively into a non-identified phase. The low-intensity peak observed between 2*θ* from 5.9 to 7.3° in all the patterns is attributed to the sample holder.

**Table S8.** Summary of Cu-based CPs: as-synthesized and obtained by transformations induced by solvent adsorption or exchange.

|  | *Procedure* | |
| --- | --- | --- |
| CP | *from synthesis* | *from Transformation* |
| **1-TCM^SC^** | Three-layer crystallization method:  -**L1** in chloroform  -THF (intermediate layer)  -CuCl_2_·2H_2_O in MeOH | - |
| **1-MeCN^SC^** | Three-layer crystallization method:  -**L1** in chloroform  -THF (intermediate layer)  -CuCl_2_·2H_2_O in MeCN | - |
| **1-H_2_O^SC^** | - | Exposure of **1-TCM^SC^**to EtOH vapors for 2 weeks. |
| **1-Amorphous^Pwd^** | Fast crystallization from a solution of **L1** in TCM and CuCl_2_·2H_2_O in MeOH | - |
| **1-TCM^Pwd^** | - | Exposure of **1-Amorphous^Pwd^** to vapors of TCM for 2 weeks |
| **1-MeCN^Pwd^** | - | 1) Exposure of **1-TCM^SC^** to vapors of MeCN for 2 weeks  2) Exposure of **1-Amorphous^Pwd^** to vapors of MeCN for 2h  3) Exposure of **1-Amorphous^Pwd^** to a binary mixture of vapors of MeCN with MeOH, DCM, TCM, NB, ClBz or DCB for 2 weeks |
| **1-MeNO_2_^Pwd^** | - | 1)Exposure of **1-TCM^SC^** to vapors of MeNO_2_ for 2 weeks  2)Exposure of **1-Amorphous^Pwd^** to vapors of MeNO_2_ for 2 weeks  3) Exposure of **1-Amorphous^Pwd^** to a mixture of vapors of MeCN/MeNO_2_ for 2 weeks |
| **1-NB^Pwd^** | - | Exposure of **1-Amorphous^Pwd^** to vapors of NB for 2 weeks |
| **1-ClBz^Pwd^** | - | Exposure of **1-Amorphous^Pwd^** to vapors of ClBz for 2 weeks |
| **1-DCB^Pwd^** | - | Exposure of **1-Amorphous^Pwd^** to vapors of DCB for 2 weeks |

Methanol (MeOH), Ethanol (EtOH), acetonitrile (MeCN), nitromethane (MeNO_2_), dicholoromethane (DCM), nitrobenzene (NB), chlorobenzene (ClBz) and 1,2-dichlorobenzene (DCB).

1. C. F. Macrae, I. Sovago, S. J. Cottrell, P. T. A. Galek, P. McCabe, E. Pidcock, M. Platings, G. P. Shields, J. S. Stevens, M. Towler and P. A. Wood, *J. Appl. Cryst*., **2020**, 53, 226. [↑](#footnote-ref-1)
2. Kryachko ES, Zeegers-Huyskens T. *J. Phys. Chem. A.* **2002**,*106*(29):6832. [↑](#footnote-ref-2)
3. Pedzisa L, Hay BP. *J. Org. Chem.*. **2009**, *74*(6):2554. [↑](#footnote-ref-3)
4. (a) J. Rodriguez-Carvajal, *Phys*. *B*., **1993**, 192, 55; (b) J. Rodriguez-Carvajal, *Comm. Powder Diffr.(IUCr) Newsl*, **2001**, 26, 12. [↑](#footnote-ref-4)
5. G.S. Pawley, Unit-cell refinement from powder diffraction scans. *Applied Crystallography*, **1981**, 14(6), 357-361. [↑](#footnote-ref-5)
